# Elaborately Tuning Intramolecular Electron Transfer Through Varying Oligoacene Linkers in the Bis(diarylamino) Systems

**DOI:** 10.1038/srep36310

**Published:** 2016-11-02

**Authors:** Jing Zhang, Zhao Chen, Lan Yang, Fang-Fang Pan, Guang-Ao Yu, Jun Yin, Sheng Hua Liu

**Affiliations:** 1 Key Laboratory of Pesticide and Chemical Biology, Ministry of Education, College of Chemistry, Central China Normal University, Wuhan 430079, P.R. China; 2 Jiangxi Key Laboratory of Organic Chemistry, Jiangxi Science and Technology Normal University, Nanchang, Jiangxi 330013, P. R. China

## Abstract

The research efforts on oligoacene systems are still relatively limited mainly due to the synthetic challenge and the extreme instability of longer acenes. Herein, these two issues have been overcome through elaborative modification and the stable pentacene species has been successfully synthesized. Additionally, a series of bis(diarylamino) compounds linked by variable-length oligoacene bridges ranging from one to five fused rings (benzene (**1a**), naphthalene (**1b**), anthracene (**1c**), tetracene (**1d**) and pentacene (**1e**)) have been prepared to probe the effect of the extent of *π*-conjugation on the electron transfer properties. Compound **1c** exhibits a high planarity between the anthracyl bridge and the two nitrogen cores and the molecular packing shows a two-dimensional herringbone characteristic. Combined studies based on electrochemistry and spectroelectrochemistry demonstrate that (i) the electronic coupling across the oligoacene linkers between two diarylamine termini exponentially decrease with a moderate attenuation constant (*β*) of 0.14 Å^−1^ in these length-modulated systems and (ii) the associated radical cations [**1a**]^+^–[**1e**]^+^ are classified as the class II Robin–Day mixed-valence systems. Furthermore, density functional theory (DFT) calculations have been conducted to gain insight into the nature of electron transfer processes in these oligoacene systems.

Recently, mixed-valence (MV) compounds with two identical redox-active groups have attracted continuous attention because they are one of the most widely exploited model systems used to study intramolecular electron transfer processes and can provide important and broad prospects for constructing highly functionalized molecules with interesting electronic and photophysical properties essential for molecular devices^1^,^2^,^3^,^4^,^5^. Accordingly, numerous studies have focused on elaborately screening rigid and *π*-conjugated bridging ligands linking two redox termini to subtly tune the intramolecular electron-transfer properties in MV systems^6^,^7^,^8^,^9^,^10^.

Oligoacenes and their derivatives have recently received an increasing interest because of their potential applications in a variety of optoelectronic devices such as light-emitting diodes (LEDs), photovoltaic cells, organic thin-film field-effect transistors (OFETs) and liquid crystals^11^,^12^,^13^,^14^,^15^. We note that Frisbie and co-workers have recently elucidated the tunneling transport in molecular junctions based on oligoacenes and concluded that oligoacenes, serving as molecular wires for nanosized electronic devices, are more superior when compared with some typical *π*-conjugated oligomers such as polyacetylene, oligo(thiophene), oligo(meso-meso-linked zinc(II) porphyrin-butadiynylene), oligo(*p*-phenylethynylene) and oligo(*p*-phenylene)^16^,^17^,^18^. Although oligoacene compounds have always been one of the favorites of theoretical researchers^19^,^20^,^21^,^22^,^23^,^24^, the research efforts on such systems combining theory and experiment are still relatively limited mainly due to the synthetic challenge and the extreme instability of longer acenes, let alone the detailed and systematic comparison of their electronic transfer^25^,^26^,^27^,^28^,^29^,^30^. Accordingly, a thorough understanding and regular exploration of their electronic transfer properties, from both an experimental and theoretical perspective is of great significance for the design and development of electronic devices intimately tied to oligoacenes.

Triarylamine has been identified as an almost ideal redox center to study the electron-transfer processes due to its reversibly oxidizable superiority^31^,^32^,^33^,^34^,^35^,^36^,^37^,^38^,^39^,^40^,^41^. In this work, we have therefore set out to assemble differently fused oligoacenes ranging from benzene up to pentacene and triphenylamine (Fig. 1). As an ideal redox center, the triphenylamine moiety has been expected to act as the desirable probe for our evaluation of the electron transport in the oligoacene series. Our goal in the present study is to elaborate how a change in the extension of the fused aromatic ring will affect the redox, spectroscopic and electron transfer properties of the investigated series of compounds. We have employed a joint experimental and theoretical method involving electrochemistry, UV-vis-NIR spectroelectrochemistry, and density functional theory calculations to make an in-depth discussion on a series of diamine compounds featuring variable-length oligoacene linkers (benzene, naphthalene, anthracene, tetracene and pentacene).

## Results and Discussion

**Figure 2:**
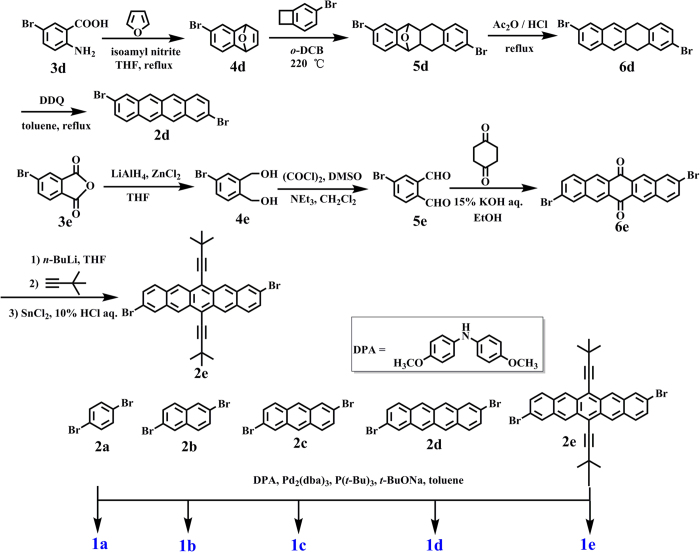
General synthetic routes to bis(diarylamino) oligoacene compounds **1a**–**1e**.

The general synthetic route used for the preparation of bis(diarylamino) oligoacene compounds **1a**–**1e** and **the** precursors **2d** and **2e** is outlined in Fig. 2. Diarylamine and the bridge precursors **2a**–**2c** were synthesized according to literature procedures. The target compounds **1a**–**1e** were prepared via a Buchwald−Hartwig coupling of their corresponding oligoacene bromides with an excess of the appropriate aniline. Pentacene is notoriously unstable and the modification or substitution of the ring is synthetically difficult. Noteworthy is that we also have made numerous attempts to improve the solubility and chemical stability of the dibromopentacene compound by varying the alkynyl substitutions at the 6,13-positions of the pentacenequinone precursor. The reason why we chose the ethynyl group at the 6,13-positions is that Miller and colleagues have instructively reported that the substitution of an electron-withdrawing ethynyl group at the 6,13-positions can reduce the HOMO–LUMO gap by stabilizing the LUMO orbital^26^. Although the linear alkyl (*n*-butyl and *n*-hexyl) ethynyl substituted dibromopentacene compounds were tentatively obtained, we finally failed to obtain the stable diamine target compounds. Ultimately, we selected the relatively bulky branched group *tert*-butyl inspired by the elegant study reported by Anthony *et al*.^42^ We improved the corresponding synthetic method using *n*-BuLi and 3,3-dimethylbut-1-yne, and obtained the desired compound **2e** in a high yield of 92%. It’s soluble in common organic solvents and the oxidative and light stability are desirable. To our great delight, the resulting target pentacene-diamine **1e** was also very soluble in common organic solvents and very stable in the solid state. The stability of **1e** in the solution state was kept for several weeks when stored in air under dark conditions. **1e** will degrade slowly when exposed to UV irradiation at 254 nm with the characteristic ^1^H NMR signals associated with the pentacene ring disappearing over time (See Fig. S1 in the Supporting Information).

**Figure 3:**
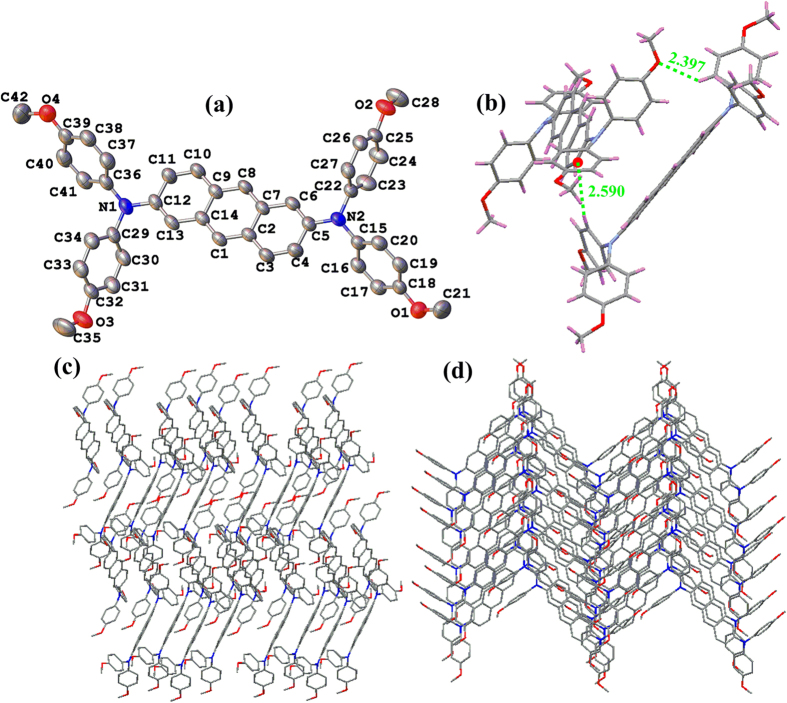
(**a**) Thermal ellipsoid plot of the X-ray structure of **1c** at 50% probability. Color scheme: carbon, grey; oxygen, red; nitrogen, blue. (**b**) Showing possible intermolecular interactions. (**c**,**d**) packing diagram of compound **1c**. Hydrogen atoms have been omitted in X-ray structure and packing views for clarity.

Suitable crystals of compound **1c** for X-ray diffraction were obtained as dark orange blocks by diffusion of CH_3_OH into a CH_2_Cl_2_ solution (Fig. 3). Important diffraction parameters are collected in Table S1. Pertinent bond lengths (Å) and bond angles (deg) are given in Table S2. There is a high planarity between the anthracyl spacer and the two adjacent nitrogen centers (Fig. 3), which allows for an optimum overlap between the *π*-orbital and the p-orbital, and further benefits electron transport across the entire system. As shown in Fig. 3, the crystallographic analysis suggests typical propeller-like arrangements for the three aromatic rings around each nitrogen center with respect to the triarylamine termini. The bond parameters including the C—C and C—N distances and the C—N—C angles, fall in the normal range of the data reported in similar systems^43^,^44^. In addition, a good coincidence between these data and the corresponding computed values can also be found in the later content. There are intermolecular C—H···*π* and hydrogen bond interactions evident between the two adjacent triarylamine moieties (Fig. 3b).

When considering the molecular packing, some additional interesting features were also found (Fig. 3c,d). The crystal packing shows a two-dimensional herringbone packing characteristic. The two adjacent stacks show a slight overlap in a head-to-tail pattern involved in the C−H···*π* and hydrogen bond interactions between the adjacent triarylamine termini. The central anthracyl ring in each layer is alternately parallel with each other, resulting the molecular packing exhibiting a staircase-like motif on the whole.

The cyclic and square-wave voltammograms (CVs and SWVs) of compounds **1a**–**1e** are shown in Fig. S2. The related data have been collected in Table 1. Reference CV plots recorded for the corresponding bromide precursors **2a**–**2d** show no redox waves in the potential interval −1.0 to +2.0 V (vs Ag/Ag^+^), while **2e** exhibits a reversible oxidation wave at 0.49 V and a reduction wave at a cathodic potential (Fig. S3). As previously reported^45^,^46^, **1a** and **1b** underwent two successive one-electron oxidations; **1c** and **1d** also displayed similar reversible anodic processes. Each of them exhibits a remarkable separation between the first two steps of the redox processes, Δ*E*_1/2_, with respect to disproportionation *K*_c_, suggesting a reasonable resonance stabilization of the positive charge and the unpaired electron in their monocationic states. It is worth noting that in the present series the values of Δ*E*_1/2_ decrease in the following sequence: 485 mV (**1a**), 375 mV (**1b**), 295 mV (**1c**) and 122 mV (**1d**) with a decrease in the delocalization extent in the radical cations (*vide infra*). Inversely, the first anodic potentials increase from **1a** (−0.200 V) over **1b** (0.094 V) and **1c** (0.138 V) to **1d** (0.290 V) and this trend is well reproduced by the density functional theory (DFT)-calculated HOMO energies (vide infra). Although the values of Δ*E*_1/2_ are not completely reliable to evaluate the electronic coupling in MV species, both of them are closely related.

Exceptionally, compound **1e** with the longest pentacene linker exhibits three steps of oxidation processes. The abnormal cathodic shift in its first oxidation potential relative to **1d** was primarily due to the *π*-donating influence of the two *tert*-butyl alkyne substituents. When compared with its precursor **2e** (Fig. S3), the first two steps may be involved in the oxidations of the two amine termini and the third one is more associated with the pentacene bridge, which is well confirmed by the following spectroelectrochemical studies.

**Figure 4:**
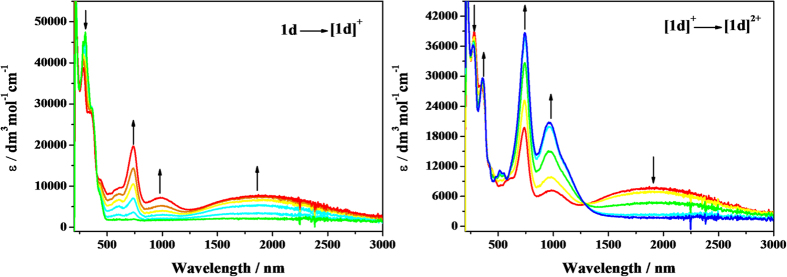
UV-Vis-NIR spectral changes recorded during the oxidation **1d → **[**1**]^+^ (left) and [**1**]^+^**→ **[**1**]^2+^ (right) in CH_2_Cl_2_/10^−1 ^M *n*-Bu_4_NPF_6_ at 298 K within an OTTLE cell.

**Figure 5:**
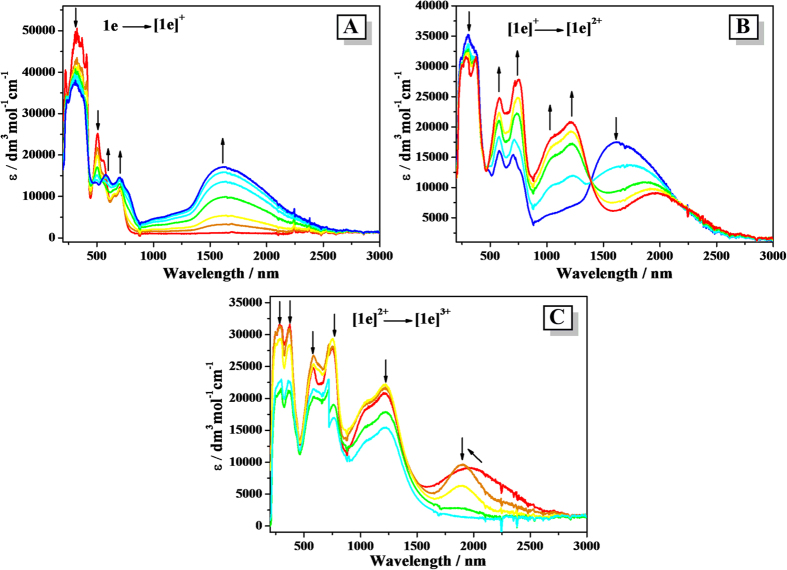
UV-Vis-NIR spectral changes recorded during the oxidation **1e → **[**1e**]^+^ (A), [**1e**]^+^** → **[**1e**]^2+^ (B) and [**1e**]^2+^** → **[**1e**]^3+^ (C) in CH_2_Cl_2_/10^−1 ^M *n*-Bu_4_NPF_6_ at 298 K within an OTTLE cell.

To gain more insight into the electronic structure corresponding to the oxidized species of **1a**–**1e**, the potential-dependent UV-vis-NIR absorption spectra of **1a**–**1e** have been measured. Spectral changes in the UV-vis-NIR region are shown in Figs 4 and 5 (**1d** and **1e**) and S4–S6 (**1a**–**1c**) with the relevant data listed in Table 2.

In the neutral forms, compounds **1a**–**1e** all show intense aromatic absorptions and the bands shifted to lower-energy with an increase in the bridging length. The sensitivity of the spectra to the degree of *π*-conjugation of the acene-based linkers was consistent with the significant contributions to the relevant HOMO orbitals (*vide infra*). Upon the stepwise oxidation to the corresponding monocationic forms [**1a**]^**+**^–[**1e**]^**+**^, the original intense absorptions gradually decreased and strong absorptions appeared simultaneously in the NIR region, accompanied by the development of new bands at around 600–800 nm, which is characteristic for triarylamine radical cations (the N^•+^-localized transition)^47^,^48^,^49^. Distinctively, the NIR absorption bands of [**1a**]^**+**^–[**1c**]^**+**^ are relatively narrow and those of [**1d**]^**+**^ and [**1e**]^**+**^ are very broad, which cover the whole biomedical and telecommunication regions (Fig. S7).

Further oxidation to [**1a**]^2**+**^–[**1d**]^2**+**^ led to the complete disappearance of the absorption bands in the NIR region, while the N^•+^ radical bands were red-shifted and continue to rise drastically. Similar absorption spectral changes can be observed during the double-oxidation process of **1e**, however, the newly generated absorptions were more complicated. When monitoring the third step of the oxidation of compound **1e**, the original absorptions belonging to [**1e**]^2+^ all decreased and no any other new band appeared with the ultimate oxidation of the pentacene bridge.

**Figure 6:**
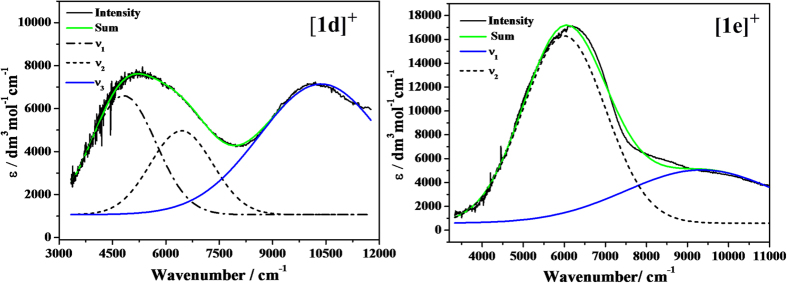
Deconvolutions of the NIR absorptions of [**1d**]^+^ (left) and [**1e**]^+^ (right) as recorded during the spectroelectrochemical measurements into Gaussian-shaped bands.

Analogous to the intervalence charge-transfer (IVCT) absorptions observed in the previously reported strongly coupled bis(diarylamino) MV systems^49^,^50^,^51^. [**1a**]^+^–[**1c**]^+^ exhibit similar intense and asymmetric NIR absorptions. The associated asymmetry has been elucidated in terms of the coupling of the electron-transfer reaction coordinated to the symmetric vibrational modes^49^. As shown in Table 3, the asymmetry of the IVCT bands of [**1a**]^+^–[**1c**]^+^ can be characterized by 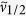
(high)/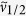
(low), where 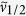
(high) and 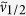
(low) are twice the half-widths of the high- and low-energy side of the band, respectively^46^. It’s clear that each value of 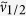
(high)/Δ
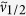
(calc) for [**1a**]^+^–[**1c**]^+^ was approximated to 1, showing that the high-energy side is expected to fit well to the Gaussian curve^46^. While the spectra for [**1d**]^+^ and [**1e**]^+^ each shows a broad absorption band, which obviously corresponds to overlay of three and two sub-bands, respectively, by deconvolution of the Gaussian function (Fig. 6). Their higher energy and broader sub-bands (blue lines in Fig. 6) can be assigned to intervalence charge transfer (IVCT).

The width of the IVCT bands observed for [**1a**]^+^–[**1e**]^+^ can be evaluated through the comparison between the values of Δ
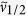
(obs) and those predicted from Hush theory for class-II MV species, Δ
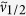
(calc), calculated by equation (1)^51^ Δ
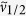
(calc) = [2310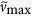]^1/2^. Apparently, the observed bandwidths at half height (Δ
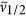
(obs)) for [**1a**]^+^–[**1e**]^+^ are narrower than those calculated using Hush’s theory.

**Figure 7:**
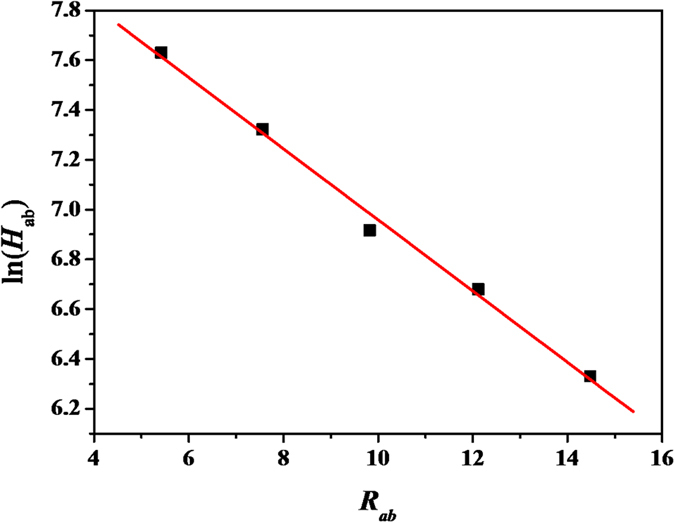
Distance dependence plot of ln(*H*_ab_) as a function of *R*_ab_ (Å) from data in Table 3. The data was fitted to a linear equation with a slope of −0.14 Å^−1^ and adjusted *R*^2^ of 0.995.

The intervalence transition is basically regarded as a special case of charge transfer transition, in which the reduced site acts as the electron donor and the oxidized site as the acceptor^52^. The electronic coupling, *H*_ab_, is responsible for the mixing of the two electronic states as well as the intensity of the intervalence transition. Accordingly, *H*_ab_, for both class-II and III species, can be determined estimated from characteristics of the IVCT using the Hush expression^53^,^54^ 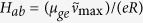 [equation (2)]. This expression makes no implicit assumption about the shape of the NIR band^46^, where *R* is the effective separation between the donor and acceptor (diabatic states), and the determination of its “true” value is still a problem. We have used the N−N distance to estimate *H*_ab_, which is generally smaller than *R*, meaning an underestimation of *H*_ab_. *e* is the elementary charge and *μ*_ge_ is the transition dipole moment associated with the transition and given, in Debye, by: 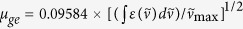 [equation (3)], where 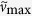
and *ε*(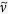) are in cm^−1^ and M^−1 ^cm^−1^, respectively. In the case of symmetric Gaussian IVCT bands, equations (2) and (3) are often combined to give equation (4): 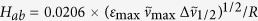. Alternatively, if the radical cations are strongly coupled class-III MV systems, the *H*_ab_ terms can be calculated directly from equation (5): *H*_ab_ = 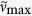/2. The appropriate parameters have been summarized in Table 3, which reveal some interesting trends associated with the molecular structure. The calculated *H*_ab_ values of [**1a**]^+^–[**1c**]^+^ from equation (2) are much smaller than those obtained from equation (5), which eliminates the classification of fully delocalized class-III species for the mono-oxidized forms [**1a**]^+^–[**1c**]^+^. Actually, the most delocalized [**1a**]^+^ is still a class II compound just on the class-II/III borderline consistently as has been clarified^46^. We should note that the values of *H*_ab_, even for the tetracene and pentacene radical cations, are still remarkable and the two systems still behave as a class II system according to the scheme introduced by Robin and Day^55^. Comparatively, the values obtained for the electronic coupling *H*_ab_ of [**1a**]^+^–[**1e**]^+^ generally decrease in the order: [**1a**]^+^ > [**1b**]^+^ > [**1c**]^+^ > [**1d**]^+^ > [**1e**]^+^, with an increase in the bridging lengths. We were more interested in the distance dependence of the electronic coupling. The values of *H*_ab_ versus *R* for this series of oligoacene-bridged systems based on the results in Table 3 are shown in Fig. 7. A good (*R*^2^ = 0.995) linear correlation of ln(*H*_ab_) versus *R* can be observed with a *β* value of 0.14 and *H*_0_ = 4394 cm^−1^. The linear correlation confirms that [**1e**]^+^ still belongs to the class II MV system and provides good evidence for a superexchange electron transfer mechanism^52^. More importantly, the *β* value was comparable to that found for the oligophenylene-bridged ruthenium-amine series (*β* = 0.14 Å^−1^)^56^ and polyyn-diyl bridged diruthenium systems (*β* = 0.12 and 0.15 Å^−1^)^3^, and was slightly larger than that found for oligofuran- (0.066 Å^−1^) and thiophene-bridged diferrocenyl systems (0.070 Å^−1^)^52^. However, it’s smaller than that observed for similar single-bond linked oligophenylene bis-triarylamine systems (0.32 Å^−1^)^46^ and much smaller than those observed in biomacromolecule systems^57^.

DFT calculations were performed using the BLYP35^58^ functional on the representative [**1c**]^*n*+^–[**1e**]^*n*+^ (*n* = 0 and 1) molecules to aid the description of the electronic characteristics of this series of compounds. The basis set employed here was 6-31G*. In order to account for solvent effects, the conductor polarizable continuum model (CPCM) in CH_2_Cl_2_ was employed for the ground state structure optimizations and analyses of [**1c**]^*n*+^–[**1e**]^*n*+^ (*n* = 0 and 1) as well as in the time-dependent (TD) DFT calculations of their electronic excitation energies.

**Figure 8:**
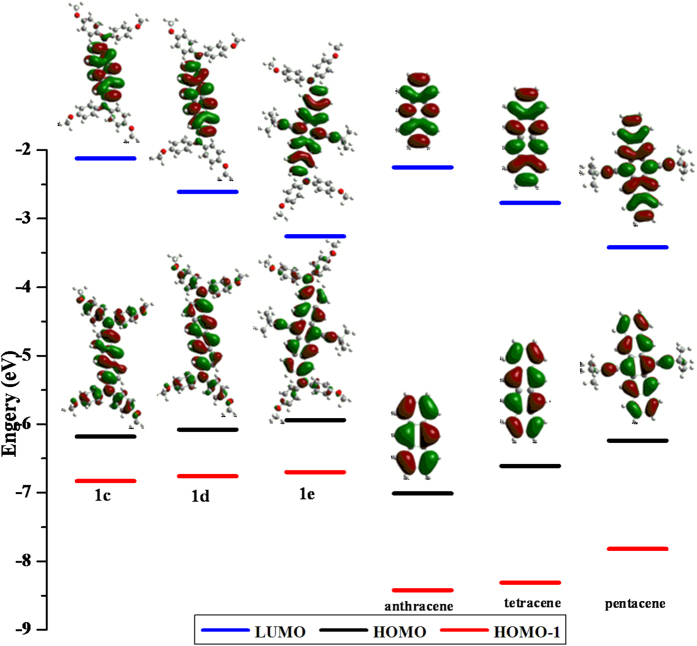
Selected BLYP35/6-31G*-derived molecular orbital energies and profiles for compounds **1c**–**1e** and corresponding bridge components. Blue, black and red lines represent LUMO, HOMO and HOMO-1, respectively.

As shown in Fig. 8, the calculation results obtained for the neutral states **1c**–**1e** display that the HOMO orbitals were all delocalized throughout the entire *π*-conjugated frameworks, while the LUMOs were mainly localized on the oligoacene fragments. By contrast, the contribution of the bridging ligand to the HOMO orbital increases with an increasing number of fused rings from **1c** to **1d** and **1e**, signifying a decreasing molecular delocalization in the present system, which was consistent with the results of the above electronic coupling assessment. Moreover, we have noticed that similar in-phase and out-of-phase orbital interactions between the HOMO-1 of the oligoacene linker and the two N 2p orbitals lead to the delocalized distribution of the HOMO across the whole molecule of **1c**–**1e** (Fig. 8 and S8)^59^. More importantly, the HOMO level was up-shifted and the LUMO was down-shifted significantly from **1c** to **1d** and **1e**. This demonstrates that the energy gap between the HOMO and LUMO for **1c**–**1e** becomes smaller and ultimately may lead to the electrons being more easily excited and thus, beneficial for absorbing longer wavelength light. The above-calculated results match extremely well with the experimental observations found in the spectral investigations.

We were then interested in comparing the geometric characteristics of the neutral and cationic states. As depicted in Fig. S9, the DFT results suggest that **1c**–**1e** all undergo similar geometric changes upon oxidation. The oxidations result in two amino N–C_bridge_ bonds shortening and the four terminal N–C_aryl_ bonds lengthening, and two benzene rings of amine center getting closer to each other, which signifies the varying degrees of delocalization in the present systems.

According to the results of the TD-DFT calculations (Fig. S10), the simulations of the absorption in the visible and NIR regions for the radical cations [**1c**]^+^–[**1e**]^+^ are basically consistent with the observations recorded in the corresponding experimental spectrum. The associated singly occupied orbital (β-HOSO) corresponding to the electronic excitation in the NIR region for each case was similar to that of the homologous HOMO-1 orbital in the neutral state, which all weigh heavily on two terminal amines.

## Conclusions

In summary, we have presented a series of bis(diarylamino) compounds linked by oligoacene bridges (benzene, naphthalene, anthracene, tetracene and pentacene) in this contribution. The electronic coupling throughout the oligoacene linkers between the two diarylamine terminals was persistent and a moderately exponential decrease with an attenuation constant (*β*) of 0.14 Å^−1^ was estimated in this extended series. The radical monocations [**1a**]^+^–[**1e**]^+^ were reasonably classified as the Robin–Day class II mixed-valence systems. As demonstrated by the DFT calculations, the decreasing molecular delocalization with the extension of the fused aromatic ring made a good interpretation for the electronic coupling decay between two amine cores. The presented electrochemical and spectroelectrochemical properties suggest that the oligoacenes are promising candidates for applications as molecular wires. More importantly, this work provides insights needed to design and explore new molecular wires.

## Methods

### Physical Measurements

^1^H and ^13^C spectra were collected on a Varian Mercury Plus 400 spectrometer (400 MHz). ^1^H and ^13^C NMR chemical shifts are relative to TMS. Electron impact ionization (EI) mass spectra were carried on Trace MS 2000. HRMS were obtained on an IonSpec FT-ICR mass spectrometer with ESI resource. Elemental analyses (C, H, N) were performed with a Vario ElIII Chnso instrument. The electrochemical measurements were performed on a CHI 660C potentiostat (CHI USA). A three-electrode single-compartment cell was used for the solution of compounds and supporting electrolyte in dry CH_2_Cl_2_. The solution was deaerated by argon bubbling on a frit for about 10 min before the measurement. The analyte and electrolyte (*n*-Bu_4_NPF_6_) concentrations were typically 2 × 10^−4^ and 10^−1 ^mol dm^−3^, respectively. A pre-polished 500-μm diameter platinum disk working electrode, a platinum wire counter electrode, and an Ag wire pseudoreference electrode were used. Ferrocene was used as the internal potential reference. Spectroelectrochemical experiments at room temperature were performed with an airtight optically transparent thin-layer electrochemical (OTTLE) cell (optical path length of ca 200 μm) equipped with a Pt minigrid working electrode and CaF_2_ windows. The cell was positioned in the sample compartment of a Shimadzu UV-3600 UV-vis-NIR spectrophotometer. The controlled-potential electrolyses were carried out with a CHI 660C potentiostat. The concentration of samples was ca 2 × 10^−3 ^mol dm^−3^. Dry 10^−1 ^M *n*-Bu_4_NPF_6_ was used as the supporting electrolyte.

### Crystallographic Details

Single crystal of compound **1c** suitable for X-ray analysis was grown by slow diffusion of methanol into a solution of dichloromethane. Crystals with approximate dimension of 0.20 × 0.20 × 0.10 mm^3^ for **1c** was mounted on glass fibers for diffraction experiments. Intensity data were collected on a Nonius Kappa CCD diffractometer with Mo Kα radiation (0.71073 Å) at room temperature. The structures were solved by direct method with SHELXT and refined by full-matrix least-squares methods using the OLEX2, which utilizes the SHELXL2014 module. All non-H atoms were refined anisotropically. The hydrogen atoms were placed in ideal positions and refined as riding atoms. Further crystal data and details of the data collection are summarized in Table S1. Selected bond distances and angles are given in Table S2. CCDC 1493451 for **1c**.

### Computational Details

DFT calculations were performed with the Gaussian 09 program, at the BLYP35/6-31G* levels of theory. Geometry optimizations were performed without any symmetry constraints, and frequency calculations on the resulting optimized geometries showed no imaginary frequencies. Electronic transitions were calculated by the time-dependent DFT (TD-DFT) method. The MO contributions were generated using the Multiwfn package and plotted using GaussView 5.0. The solvation effects in dichloromethane are included for a part of the calculations with the conductor-like polarizable continuum model (CPCM).

## Additional Information

**How to cite this article**: Zhang, J. *et al*. Elaborately Tuning Intramolecular Electron Transfer Through Varying Oligoacene Linkers in the Bis(diarylamino) Systems. *Sci. Rep*. **6**, 36310; doi: 10.1038/srep36310 (2016).

**Publisher’s note:** Springer Nature remains neutral with regard to jurisdictional claims in published maps and institutional affiliations.

## Supplementary Material

Supplementary Information

## Figures and Tables

**Figure 1:**
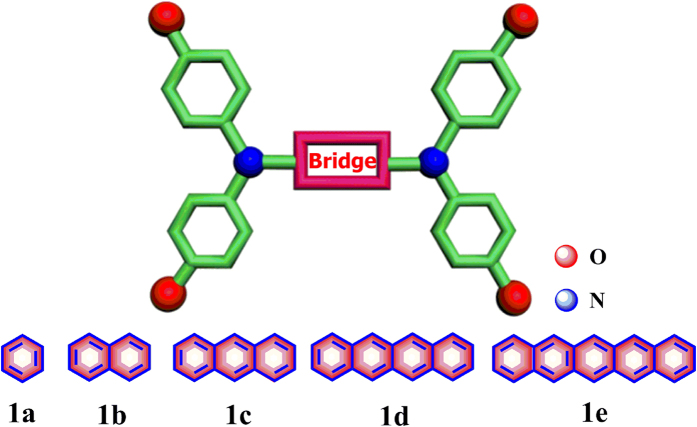
Studied series of bis(diarylamino) compounds **1a**–**1e** bridged by oligoacene ligands.

**Table 1:** Electrochemical data for compounds 1a–1e^*a*^.

Compound	*E*_1/2_ (1) (V)	*E*_1/2_ (2) (V)	*E*_1/2_ (3) (V)	Δ*E*_1/2_ (mV)	*K*_c_^*b*^
1a	−0.200	0.285	—	485	1.6 × 10^8^
1b	0.094	0.469	—	375	2.2 × 10^6^
1c	0.138	0.433	—	295	9.7 × 10^4^
1d	0.290	0.412	—	122	1.2 × 10^2^
1e	0.061	0.339	0.534	—	—
2e	0.490	—	—	—	—

^*a*^The anodic potentials are referenced against the standard ferrocene/ferrocenium (Fc/Fc^+^) redox couple; *E*_1/2_ (Fc/Fc^+^) = +0.43 V vs Ag/AgCl. ^*b*^ The comproportionation constants, *K*_c,_ were estimated using the expression *K*_c_ = exp(Δ*E*/25.69 mV) at 298 K.

**Table 2:** UV-vis-NIR electronic absorption of compounds 1a–1e and their oxidation products in dichloromethane/*n*-Bu_4_NPF_6_.

Compound	*λ*_max_ (nm) (*ε*_max_ × 10^−4^ (dm^3 ^mol^−1 ^cm^−1^))
1a	215 (1.77), 311 (1.50)
[1a]^+^	220 (1.40), 418 (0.89), 567 (0.37), 1040 (1.00)
[1a]^2+^	633 (1.22), 785 (1.89)
1b	278 (1.55), 347 (1.72)
[1b]^+^	274 (1.41), 397 (0.85), 511 (0.72), 603 (0.24), 1305 (1.34)
[1b]^2+^	648 (1.08), 851 (7.00)
1c	246 (2.16), 329 (2.39)
[1c]^+^	268 (1.74), 351 (1.42), 413 (0.90), 623 (0.63), 676 (0.60), 1480 (1.40)
[1c]^2+^	268 (1.75), 351 (1.00), 623 (0.74), 909 (2.07)
1d	299 (4.77)
[1d]^+^	277 (3.80), 737 (1.97), 988 (0.76), 1865 (0.80)
[1d]^2+^	272 (3.62), 357 (2.99), 742 (3.86), 967 (2.08)
1e	320 (5.03), 507 (2.54), 560 (1.89)
[1e]^+^	309 (3.74), 582 (1.53), 699 (1.46), 1613 (1.74)
[1e]^2+^	582 (2.51), 753 (2.80), 1218 (2.12), 1961 (0.91)
[1e]^3+^	288 (2.30), 368 (2.27), 721 (2.33), 1218 (1.54)

**Table 3:** Parameters from the low-energy NIR absorptions of the radical cations [1a]^+^–[1e]^+^.

	[1a]^+^	[1b]^+^	[1c]^+^	[1d]^+^	[1e]^+^
*R* [Å]^*a*^	5.42	7.56	9.81	12.12	14.48
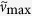 [cm^−1^] (*ε*_max_ [M^−1 ^cm^−1^])	9524 (8700)	7716 (13000)	6766 (12600)	10365 (6100)	9342 (4500)
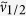 (obs)^*b*^ [cm^−1^]	3599	3091	2779	3474	3703
Δ 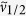 (calc)^*c*^	4690	4222	3953	4893	4645
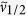 (high)/ 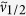 (low)^*d*^	1.67	1.42	1.81	—	—
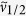 (high)/Δ 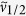 (calc)^*e*^	0.96	0.86	0.91	—	—
*μ*_ge_^*f*^ [*D*]	5.62	7.11	7.02	—	—
*H*_ab_^*g*^ [cm^−1^]	2061	1512	1009	796	561
*H*_classIII_^*h*^ [cm^−1^]	4762	3858	3383	—	—

^*a*^Evaluated by the DFT-optimized N−N geometrical distance in mono-cationic state. ^*b*^ 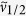
(obs) is the observed half-height bandwidth of IVCT band. ^*c*^From equation (1) at ambient temperature. ^*d*^Ratio of bandwidth on high-energy side to that on low-energy side. ^*e*^Ratio of twice the band on the high-energy side to the calculated bandwidth. ^*f*^Transition dipole moment calculated from the IVCT band using equation (3). ^*g*^The electronic coupling *H*_ab_ was calculated by using equation (2) for [**1a**]^+^–[**1c**]^+^ and equation (4) for [**1d**]^+^ and [**1e**]^+^. ^*h*^From equation (5) using the experimental values of 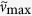.
