# Supplementary material for: Elaborately Tuning Intramolecular Electron Transfer Through Varying Oligoacene Linkers in the Bis(diarylamino) Systems
Source: Sci Rep. 2016 Nov 2;6:36310. doi: 10.1038/srep36310 (PMC5090870; doi:10.1038/srep36310)
Supplement: Supplementary Information [file srep36310-s1.pdf]

Electronic Supplementary Information (ESI)

Elaborately Tuning Intramolecular Electron Transfer Through Varying Oligoacene Linkers in the Bis(diarylamino) Systems

Jing Zhang[1,+], Zhao Chen[2,+], Lan Yang[1], Fang-Fang Pan[1], Guang-Ao Yu[1], Jun Yin[1,]* & Sheng Hua Liu[1,]*

[1] *Key Laboratory of Pesticide and Chemical Biology, Ministry of Education, College of Chemistry, Central China Normal University, Wuhan 430079, P.R. China*

[2] *Jiangxi Key Laboratory of Organic Chemistry, Jiangxi Science and Technology Normal University, Nanchang, Jiangxi 330013, P. R. China.*

* Corresponding author E-mail: yinj@mail.ccnu.edu.cn; chshliu@mail.ccnu.edu.cn

[+] These authors contributed equally to this work.

General Materials. All manipulations were carried out under an argon atmosphere by using standard Schlenk techniques, unless otherwise stated. Solvents were predried, distilled, and degassed prior to use, except those for spectroscopic measurements, which were of spectroscopic grade. The starting materials bis(4-methoxyphenyl)amine (DPA)[1], 2,8-dibromo-5,12-dihydrotetracene (**6d**)[2], 4-bromophthalaldehyde (**5e**)[3] and 2,9-dibromopentacene-6,13-dione (**6e**)[4] were prepared by the procedures described in literature methods. The target compounds **1a**[5], **1b**[6] and **1c**[7] were obtained according to literature routes.

References

1 K. Zhang, L. Wang, Y. L. Liang, S. Q. Yang, J. Liang, F. Y.Cheng and J. Chen, *Synth. Met.*, 2012, **162**, 490.

2 B. Kim, S. H. Choi, X. Y. Zhu and C. D. Frisbie, *J. Am. Chem. Soc.*, 2011, **133**, 19864.

3 J. A. Kaitz, C. E. Diesendruck and J. S. Moore, *Macromolecules*, 2013, **46**, 8121.

4 D. F. Perepichka, M. Bendikov, H. Meng and F. Wudl, *J. Am. Chem. Soc.*, 2003, **125**, 10190.

5 C. Lambert, C. Risko, V. Coropceanu, J. Schelter, S. Amthor, N. E. Gruhn and J. L. Brédas, *J. Am. Chem. Soc.*, 2005, **127**, 8508.

6 C. Lambert and G. Nöll, *J. Am. Chem. Soc.*, 1999, **121**, 8434.

7 *(a)* S. Lin, G. S. Lin, C. M. Chen and W. Shan, 2007, U.S. Patent Application No. 11/736, 967; *(b)* J. Feng, J.-Y. Shao, Z.-L. Gong and Y.-W. Zhong, *Chin. J. Org. Chem.*, 2016, DOI: 10.6023/cjoc201606020.

Syntheses of Intermediate Compounds **2d**, **6e** and **2e**

Synthesis of **2d** (2,8-dibromotetracene): 2,8-dibromo-5,12-dihydrotetracene **6d** (388 mg, 1.00 mmol) and DDQ (2,3-dicyano-5,6-dichlorobenzoquinone, 272 mg, 1.20 mmol) were dissolved in 5 mL of toluene, and the mixture was heated to 100 ℃ for 3 h. After cooling to room temperature, the precipitate was removed by filtration, and the filtrate was concentrated in vacuo and was purified by flash silica gel column chromatography (CH_2Cl_2) to obtain a mixture. The mixture was put into 10 mL of MeOH to produce a brick-red precipitate of the title compound **2d**, which was collected by filtration. Yield: 121 mg (31%). ^{1}H NMR (400 MHz, $CDCl_3$): δ 7.46 (m, 3H), 7.82-7.86 (m, 2H), 8.24 (s, 1H), 8.54-8.63 (m, 3H), 8.98 (s, 1H). EI-MS: *m/z* = 386.00 $[M]^+$; calculated exact mass = 385.91. Anal. Calcd for $C_{18}H_{10}Br_2$: C, 56.00; H, 2.61. Found: C, 56.26; H, 2.54. Note: This compound has poor solubility in many deuterated solvents, including $CDCl_3$, in which the ^{13}C NMR spectrum could not be completely recorded.

Synthesis of **6e** (2,9-dibromopentacene-6,13-dione): To a solution of **5e** (426 mg, 2.00 mmol) and 1,4-cyclohexanedione (112 mg, 1.00 mmol) in ethanol (20 mL), was added 15% KOH aqueous solution (3 mL) at room temperature. The reaction turned progressively dark brown. The precipitate was collected by filtration and washed subsequently with water, ethanol and diethyl ether, giving yellow powder of product (457 mg, 98%), which was used directly in the next step, without further purification.

Synthesis of **2e** (2,9-dibromo-6,13-bis((*tert*-butylethynyl)pentacene): To an oven-dried 100-mL round-bottom flask equipped with a stir bar and cooled to -78 ℃ under N_2 was added 2.9 mL *n*-BuLi (7.2 mmol, 2.5 M solution in hexane), followed by the dropwise addition of 1.0 mL 3,3′-dimethylbut-1-yne (8.1 mmol). This mixture

was stirred for 1 h, then dry THF (60 mL) and quinone **6e** (835 mg, 1.8 mmol) were added. The mixture was heated at 60 ℃ overnight, then quenched with 0.5 mL of water. $SnCl_2$ (0.90 g, 4.0 mmol) in 10 % HCl (2 mL) was added and the reaction solution turned deep blue. The mixture was stirred for 2 h at 60 ℃. The reaction mixture was poured into H_2O (25 mL) and extracted with CH_2Cl_2 (3 × 20 mL). The combined organic layers were dried over Na_2SO_4. The solvent was removed in vacuo. The resulting crude product was rapidly precipitated from DCM / CH_3OH to yield pure **2e** as deep blue solid (987 mg, 92%). ^1H NMR (400 MHz, $CDCl_3$): δ 1.66 (s, 18H, CH_3), 7.39 (d, J_{HH} = 8 Hz, 2H), 7.83 (d, J_{HH} = 12 Hz, 2H), 8.15 (s, 2H), 8.99 (J_{HH} = 8 Hz, 4H). ^{13}C NMR (100 MHz, $CDCl_3$): δ 29.4 ($C(CH_3)_3$), 31.5 (CH_3), 114.0, 114.1, 120.0, 125.1, 126.5, 129.1, 129.8, 130.0, 130.1, 130.3, 130.5, 132.3. HR-MS (ESI+): *m/z* calculated for $C_{34}H_{28}Br_2$ $[M]^+$: 596.0537; found: 596.0544. Anal. Calcd for $C_{34}H_{28}Br_2$: C, 68.47; H, 4.73. Found: C, 68.65; H, 4.69.

Syntheses of target bis(diarylamino) compounds **1d** and **1e**

1c (2,6-bis(*N*,*N*-di(4-methoxyphenyl)amino)anthracene, as reported in the reference[67]: ^1H NMR (400 MHz, $CDCl_3$): δ 6.82 (m, 8H), 7.06-7.08 (m, 8H), 7.12-7.21 (m, 4 H), 7.63 (d, J_{HH} = 8 Hz, 2H), 7.88 (s, 2H).

Synthesis of **1d** (2,8-bis(*N*,*N*-di(4-methoxyphenyl)amino)tetracene): To a 50 mL round-bottomed flask, anhydrous deoxygenated toluene (15 mL), tris(dibenzylideneacetone)dipalladium ($Pd_2(dba)_3$) (0.019 mmol, 17 mg), and tri(tertbutylphosphine) (0.037 mmol, 10% wt. in toluene) were added under nitrogen.

The catalyst mixture was stirred at room temperature under nitrogen for 20 minutes. Bis(4-methoxyphenyl)amine (1.85 mmol, 422 mg), **2d** (0.92 mmol, 357 mg) and sodium tert-butoxide (2.22 mmol, 214 mg) were added. The reaction mixture was heated to 90~100 ℃ for 48 h. The solvent was removed in vacuo, and the residue was purified by flash chromatography on silica gel (petroleum ether / ethyl acetate gradient 8:1). The product was precipitated from a CH_2Cl_2 solution with MeOH to give 289 mg (46%) of an orangered powder. ^1H NMR (400 MHz, $CDCl_3$): 3.81 (s, 12H, CH_3), 6.83-6.86 (m, 10H), 7.08-7.13 (m, 9H), 7.36-8.81 (m, 7H). ^{13}C NMR (100 MHz, $CDCl_3$): δ 55.6 (O-CH_3), 114.6, 125.7, 126.1, 128.2, 129.1, 140.8, 141.1, 145.7, 146.1, 147.1, 155.3, 155.5, 155.7. HR-MS (ESI+): *m/z* calculated for $C_{46}H_{38}N_2O_4$ $[M+H]^+$: 683.2904; found: 683.2889. Anal. Calcd for $C_{46}H_{38}N_2O_4$: C, 80.92; H, 5.61; N, 4.10. Found: C, 80.65; H, 5.69; N, 4.02.

Synthesis of **1e** (2,9-bis(*N*,*N*-di(4-methoxyphenyl)amino)pentacene): Using the same general procedure as for compound **1d**, compound **2e** (0.34 mmol, 200 mg), bis(4-methoxyphenyl)amine (0.75 mmol, 171 mg), sodium tert-butoxide (1.19 mmol, 114 mg), $Pd_2(dba)_3$ (0.014 mmol, 12 mg), tri(tertbutylphosphine) (0.031 mmol, 10% wt. in toluene) and anhydrous deoxygenated toluene (15 mL) were combined under nitrogen. Yield: 198 mg of a dark purple powder (66%). ^1H NMR (400 MHz, $CDCl_3$): 1.53-1.63 (m, 18H, CH_3), 3.82 (s, 12H, O-CH_3), 6.86 (d, J_{HH} = 8 Hz, 8H), 7.08-7.13 (m, 10H), 7.23 (s, 2H), 7.75-7.79 (m, 2H), 8.64 (d, J_{HH} = 4 Hz, 2H), 8.93 (s, 2H). ^{13}C NMR (100 MHz, $CDCl_3$): δ 29.3 ($C(CH_3)_3$), 31.6 (CH_3), 55.5 (O-CH_3), 112.6, 114.1, 114.5, 115.4, 116.6, 122.4, 124.5, 125.4, 125.5, 126.4, 129.0, 129.3, 140.5, 145.2,

145.3, 155.7. HR-MS (ESI+): *m/z* calculated for $C_{62}H_{56}N_2O_4$ $[M+H]^+$: 893.4324; found: 893.4321. Anal. Calcd for $C_{62}H_{56}N_2O_4$: C, 83.38; H, 6.32; N, 3.14. Found: C, 83.57; H, 6.51; N, 3.06.

Photodegradation of 1e

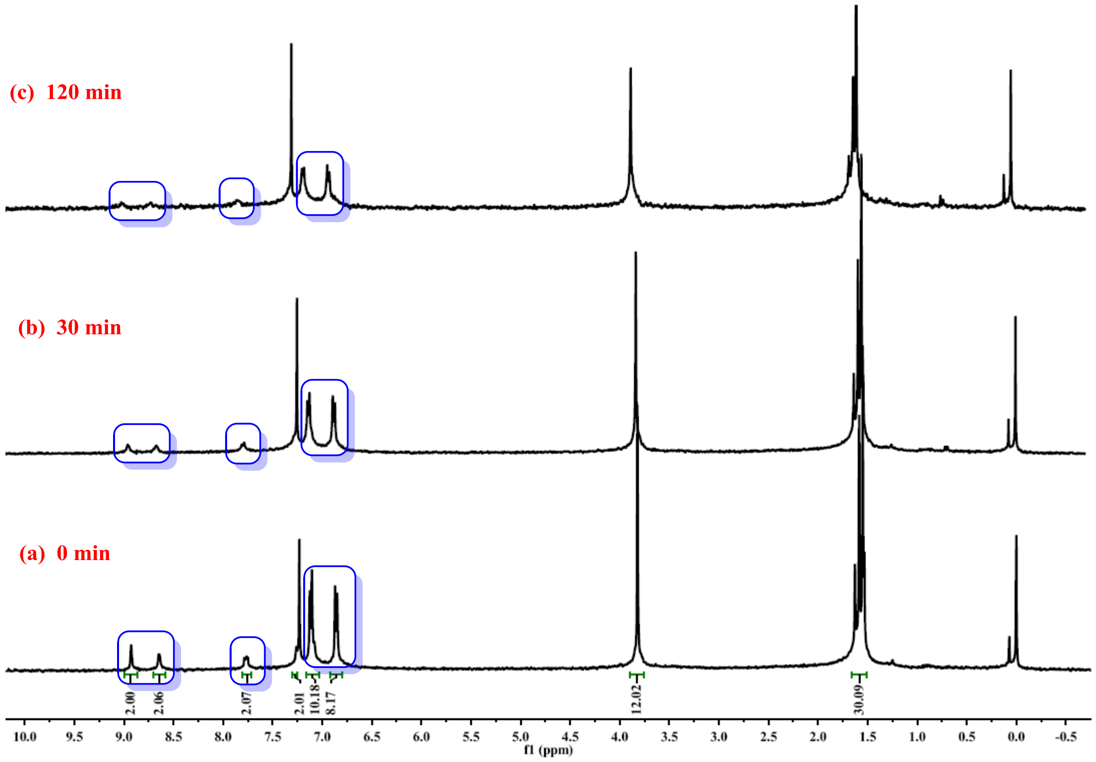

Fig. S1 ^{1}H NMR (400 M, in $CDCl_3$) spectral change associated with the photodegradation of **1e** at room temperature under UV irradiation at 254 nm in the air conditions.

Table S1. Crystal data and data collection and refinement parameters for compound **1c**.

Compound	**1c**
Formula	$C_{42}H_{36}N_2O_4$
Formula weight	632.73
Temperature (K)	298(2)
Crystal system	Monoclinic
Space group	*Cc*
a (Å)	18.493 (3)
b(Å)	11.0439 (17)
c(Å)	16.785 (3)
α (°)	90
β (°)	101.077 (2)
γ (°)	90
V (Å^3)	3364.1 (9)
Z	4
Density (calculated) (Mg/m^3)	1.249
Absorption coefficient (mm^{-1})	0.08
F(000)	1336
Crystal size (mm^3)	0.20 × 0.20 × 0.10
Theta range for data collection (°)	2.244 to 26.373
Index ranges	$-23\leq h\leq 23$, $-13\leq k\leq 13$, $-21\leq l\leq 20$
Reflections collected	13161
Independent reflections	6851 [*R*(int) = 0.025]
Max. and min. transmission	0.6778 and 0.7454
Data / restraints / parameters	6851 / 26 / 437
Goodness-of-fit on F^2	1.022
Final *R* indices [$I>2\sigma(I)$]	*R*1 = 0.0413, w*R*2 = 0.1094
R indices (all data)	*R*1 = 0.0614, w*R*2 = 0.1237
Largest diff. peak and hole (e Å^{-3})	0.14 and -0.17

Table S2. Selected bond lengths (Å) and angles (deg) in the crystal structure of compound **1c**.

Bond lengths (Å)			
O1—C18	1.369 (4)	C11—C12	1.428 (5)
O1—C21	1.414 (5)	C12—C13	1.353 (4)
O2—C25	1.362 (4)	C13—C14	1.431 (4)
O2—C28	1.414 (6)	C15—C16	1.396 (5)
O3—C32	1.366 (4)	C15—C20	1.383 (4)
O3—C35	1.411 (6)	C16—C17	1.367 (5)
O4—C39	1.370 (5)	C17—C18	1.387 (5)
O4—C42	1.407 (6)	C18—C19	1.376 (5)
N1—C12	1.409 (4)	C19—C20	1.380 (5)
N1—C29	1.437 (4)	C22—C23	1.364 (5)
N1—C36	1.428 (5)	C22—C27	1.373 (4)
N2—C5	1.401 (4)	C23—C24	1.374 (5)
N2—C15	1.422 (4)	C24—C25	1.372 (5)
N2—C22	1.440 (4)	C25—C26	1.372 (5)
C1—C2	1.392 (4)	C26—C27	1.384 (5)
C1—C14	1.385 (4)	C29—C30	1.385 (5)
C2—C3	1.419 (5)	C29—C34	1.372 (4)
C2—C7	1.443 (4)	C30—C31	1.374 (5)
C3—C4	1.358 (5)	C31—C32	1.386 (5)
C4—C5	1.431 (5)	C32—C33	1.366 (5)
C5—C6	1.364 (5)	C33—C34	1.389 (5)
C6—C7	1.416 (4)	C36—C37	1.385 (5)
C7—C8	1.387 (4)	C36—C41	1.382 (5)
C8—C9	1.397 (4)	C37—C38	1.372 (5)
C9—C10	1.412 (4)	C38—C39	1.379 (5)
C9—C14	1.439 (4)	C39—C40	1.376 (5)
C10—C11	1.347 (5)	C40—C41	1.377 (6)
**Bond angles (deg)**			
C18—O1—C21	117.6 (3)	C6—C7—C2	118.9 (3)
C25—O2—C28	117.4 (3)	C8—C7—C2	118.0 (3)
C32—O3—C35	117.7 (3)	C8—C7—C6	123.0 (3)
C39—O4—C42	118.3 (4)	C7—C8—C9	122.6 (3)
C12—N1—C29	119.3 (3)	C23—C22—N2	121.5 (3)
C12—N1—C36	120.1 (3)	C23—C22—C27	118.3 (3)

C36—N1—C29	117.0 (3)	C27—C22—N2	120.2 (3)
C5—N2—C15	120.8 (3)	C22—C23—C24	122.3 (3)
C5—N2—C22	120.1 (3)	C25—C24—C23	119.4 (3)
C15—N2—C22	115.8 (3)	O2—C25—C24	124.7 (3)
C14—C1—C2	122.6 (3)	O2—C25—C26	116.4 (3)
C1—C2—C3	123.3 (3)	C26—C25—C24	118.9 (3)
C1—C2—C7	119.3 (3)	C25—C26—C27	121.1 (3)
C3—C2—C7	117.5 (3)	C22—C27—C26	119.9 (3)
C4—C3—C2	121.7 (3)	C30—C29—N1	120.3 (3)
C3—C4—C5	121.2 (3)	C34—C29—N1	121.1 (3)
N2—C5—C4	118.5 (3)	C34—C29—C30	118.6 (3)
C6—C5—N2	123.0 (3)	C31—C30—C29	120.4 (3)
C6—C5—C4	118.4 (3)	C30—C31—C32	120.3 (3)
C5—C6—C7	122.3 (3)		

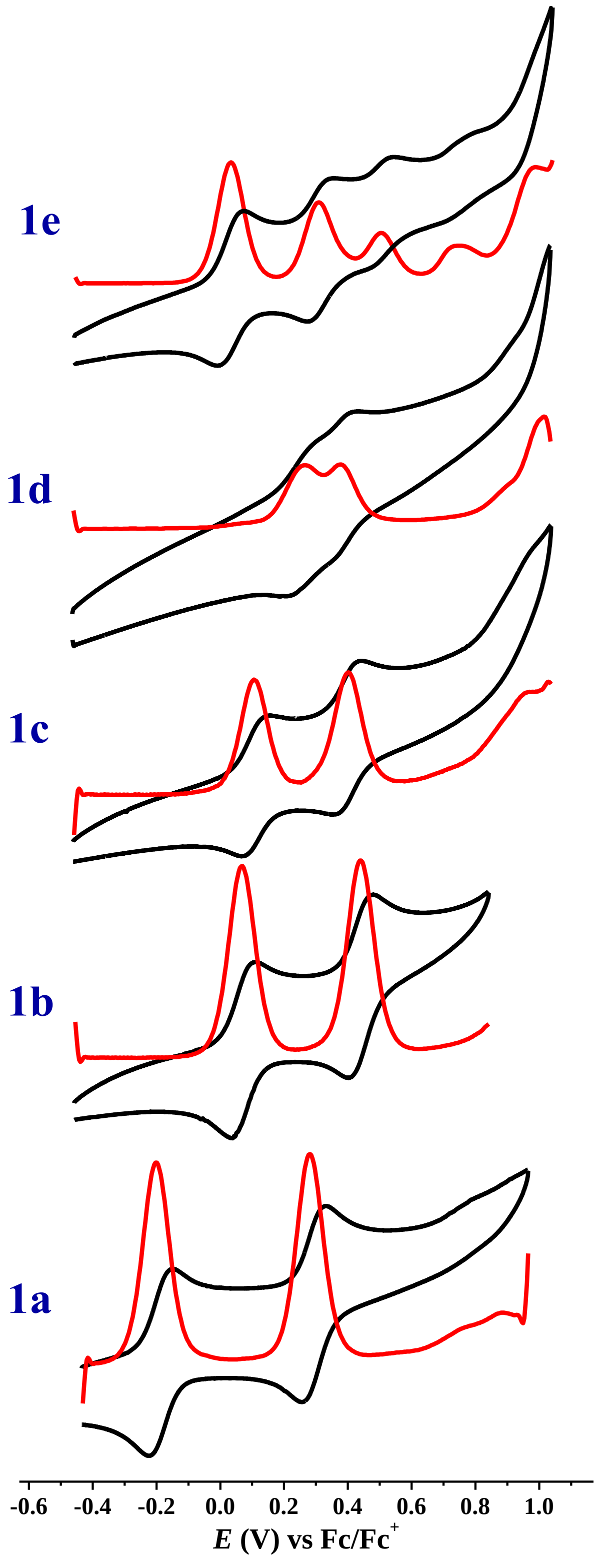

Fig. S2 Cyclic voltammograms (CV, black lines) of compounds **1a-1e** in CH_2Cl_2/ *n*-Bu_4NPF_6 at 100 mV s^{-1} and corresponding square-wave voltammograms (SWV, red lines) at f = 10 Hz (t_p = 25 mV).

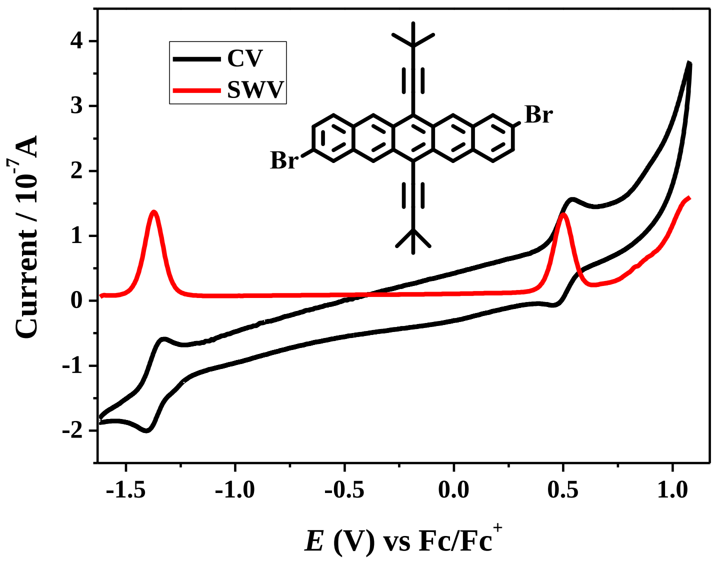

Fig. S3 Cyclic voltammogram (CV, black line) and square-wave voltammogram (SWV, red line) of **2e** in CH_2Cl_2/ *n*-Bu_4NPF_6 at 0.1 V s^{-1} (t_p = 25 mV).

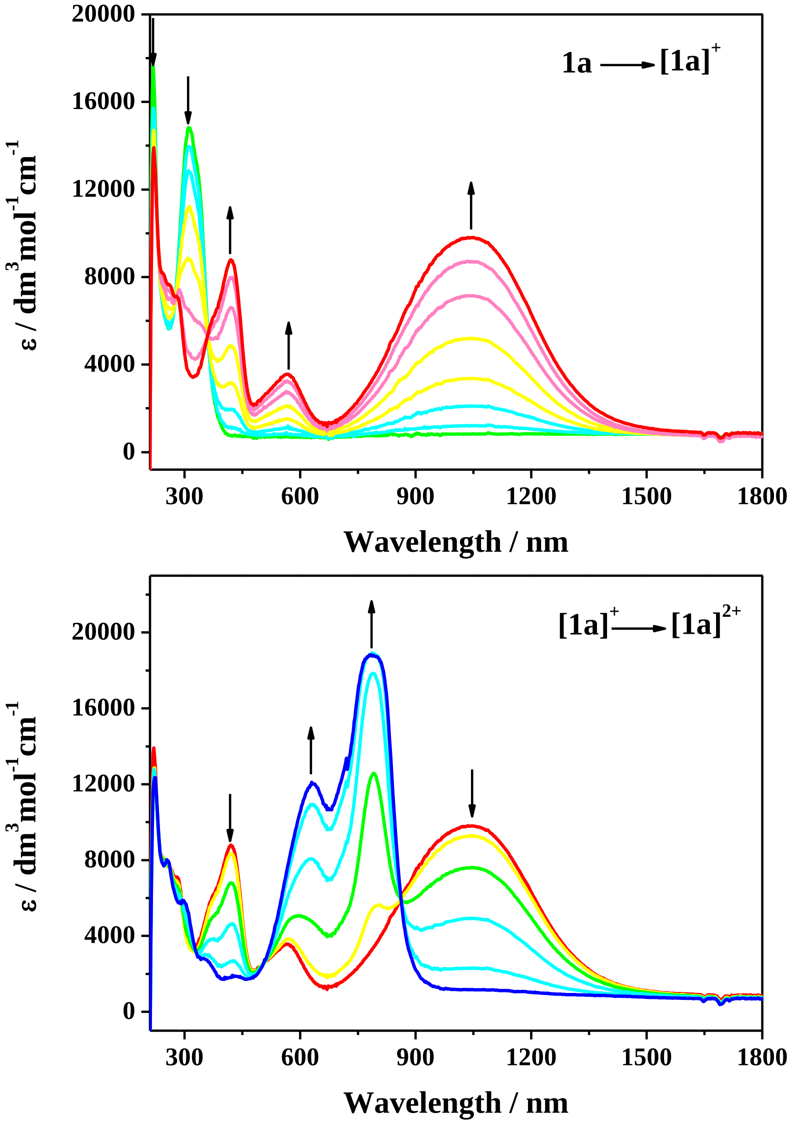

Fig. S4 UV-vis-NIR spectral changes recorded during the oxidation **1a**→[**1a**]$^+$ (top) and [**1a**]$^+$→[**1a**]$^{2+}$ (bottom) in $CH_2Cl_2/10^{-1}$ M *n*-Bu_4NPF_6 at 298 K within an OTTLE cell.

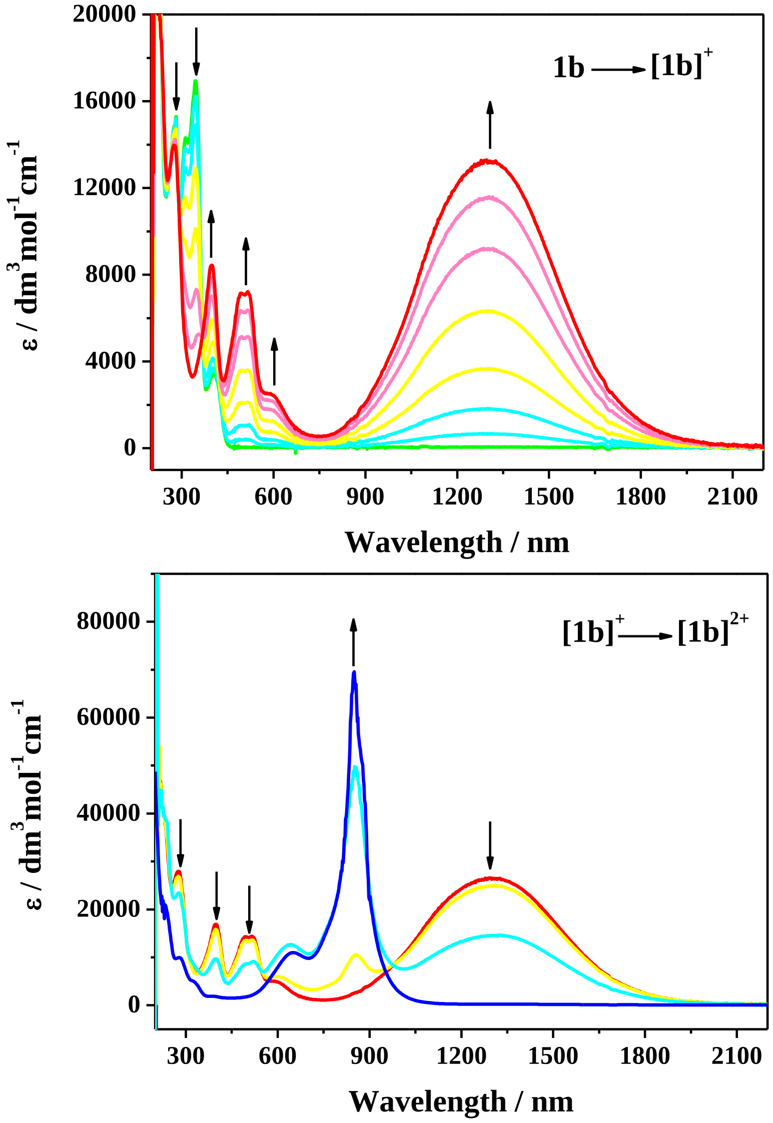

Fig. S5 UV-Vis-NIR spectral changes recorded during the oxidation **1b**→[**1b**]$^+$ (top) and [**1b**]$^+$→[**1b**]$^{2+}$ (bottom) in $CH_2Cl_2/10^{-1}$ M *n*-Bu_4NPF_6 at 298 K within an OTTLE cell.

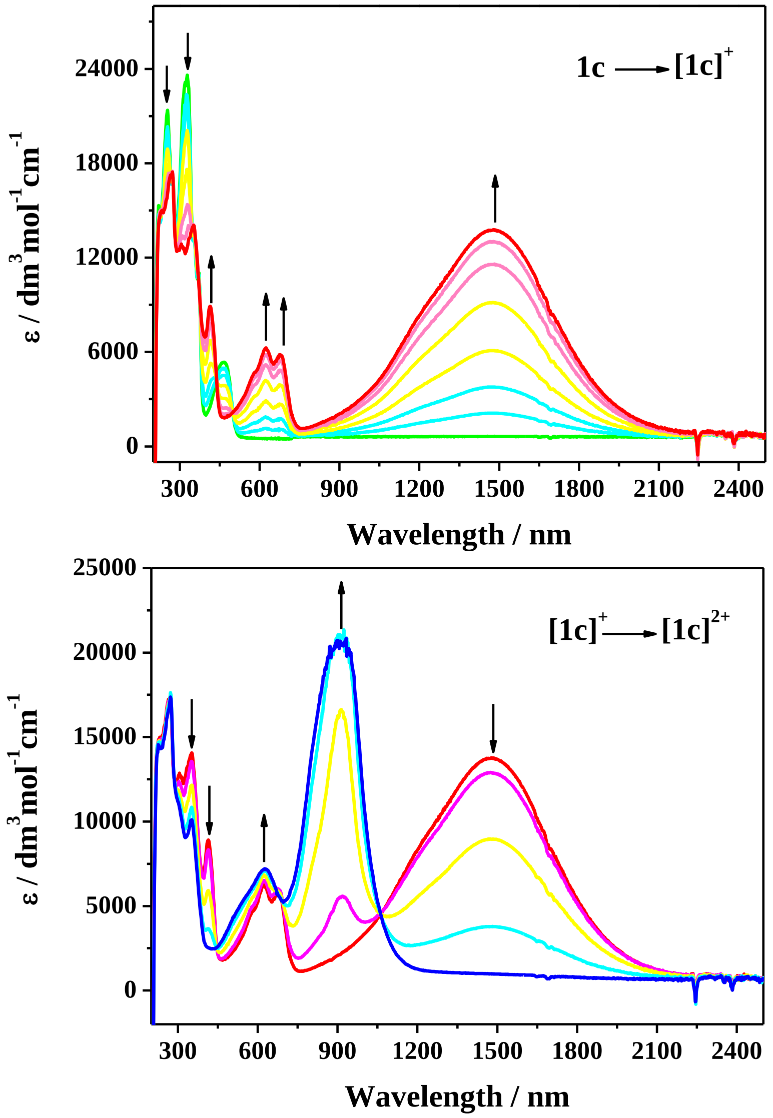

Fig. S6 UV-Vis-NIR spectral changes recorded during the oxidation **1c**→[**1c**]$^+$ (top) and [**1c**]$^+$→[**1c**]$^{2+}$ (bottom) in $CH_2Cl_2/10^{-1}$ M *n*-Bu_4NPF_6 at 298 K within an OTTLE cell.

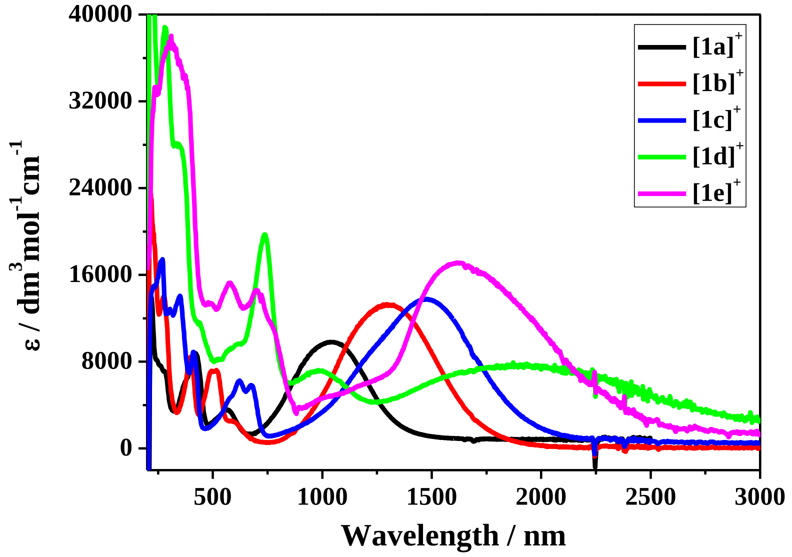

Fig. S7 Overlay of UV−vis−NIR absorption spectra for the one-electron oxidized derivatives [**1a**]$^+$–[**1e**]$^+$. All spectra were recorded in 0.1 M *n*-Bu_4NPF_6 dichloromethane electrolyte at room temperature following controlled potential electrolysis.

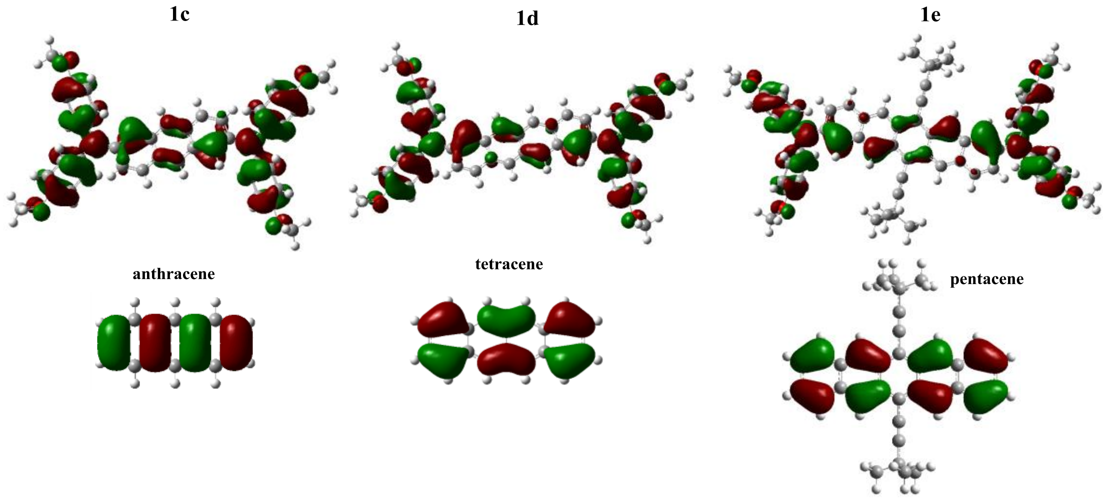

Fig. S8 The BLYP35/6-31G*-derived HOMO-1 molecular orbital profiles for compounds **1c**–**1e** and corresponding bridge components.

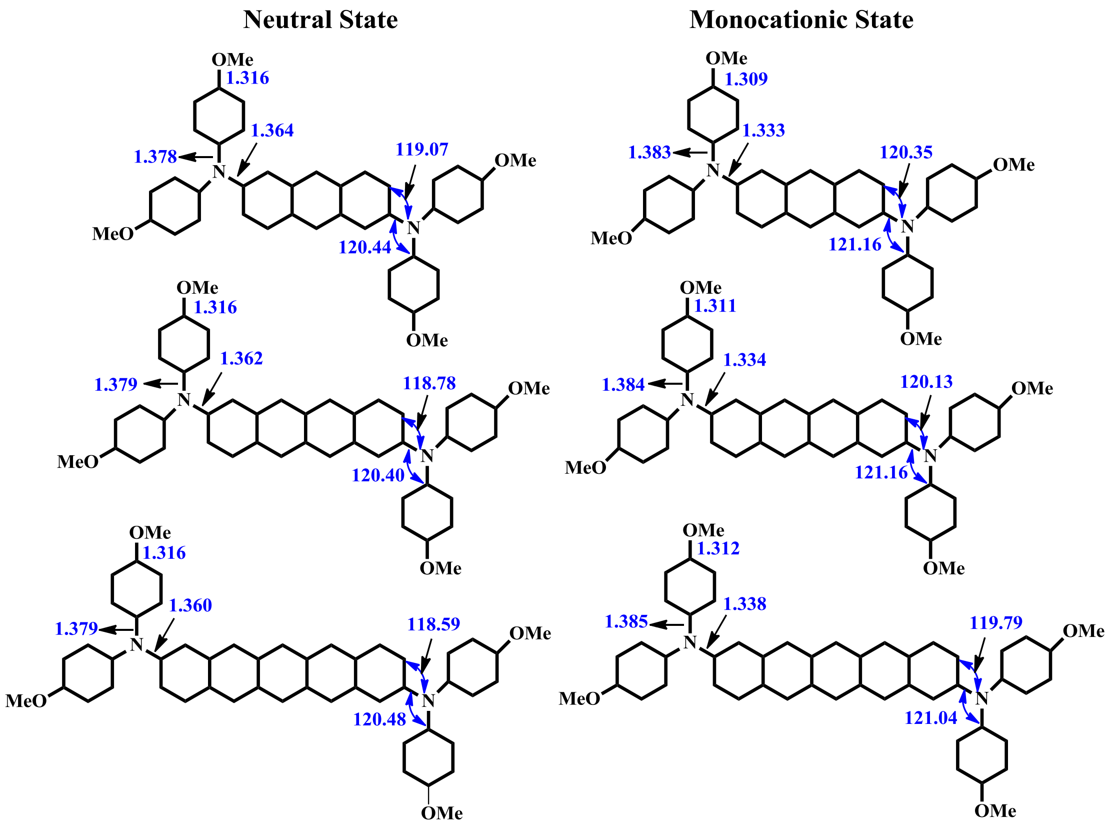

Fig. S9 Schematic diagrams of structural parameters (bond length [Å] and angle [°]) in neutral (left) and cationic (right) states of compounds **1c**–**1e** determined by DFT calculations.

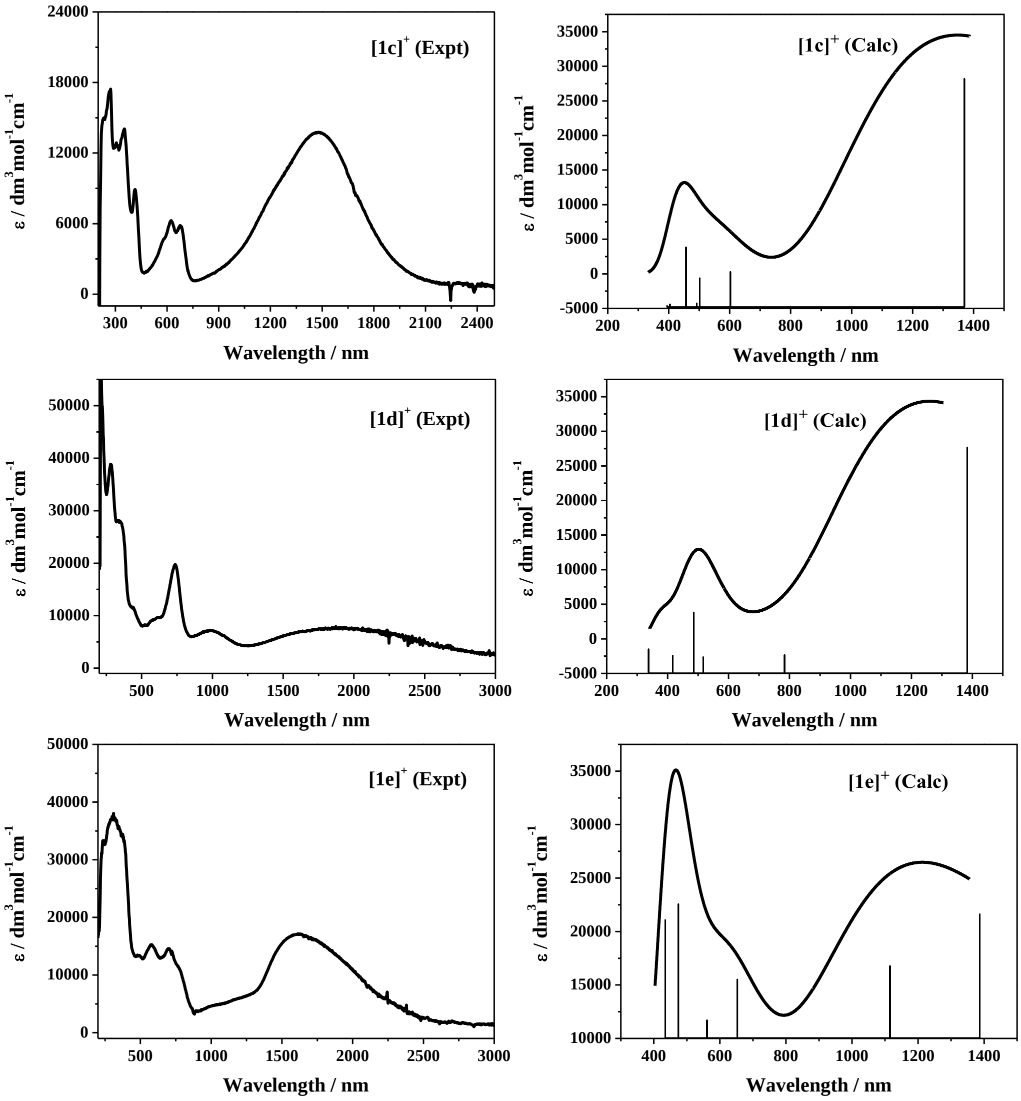

Fig. S10 Comparison between the experimental (left) and TD-DFT-calculated (right, obtained by applying a Gaussian broadening) vis−NIR spectra of the monocations [**1c**]$^+$–[**1e**]$^+$.

NMR Spectra and Mass Spectra

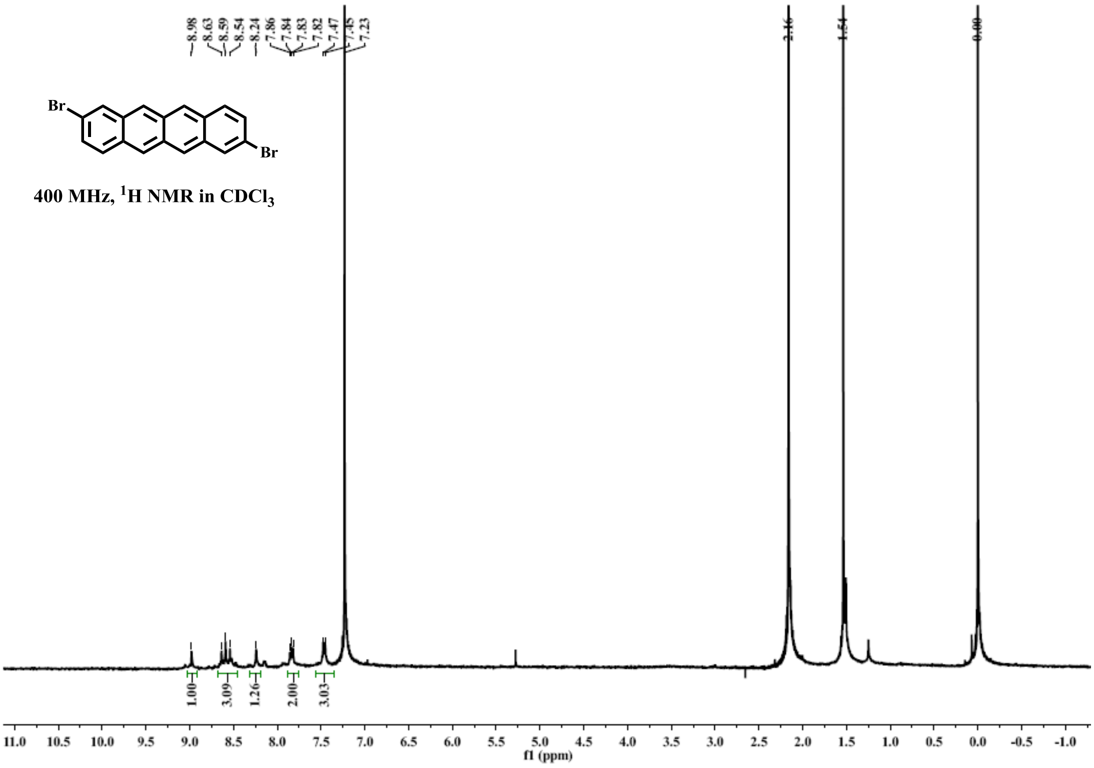

Fig. S11 ^{1}H NMR of compound **2d** (400 MHz, $CDCl_3$)

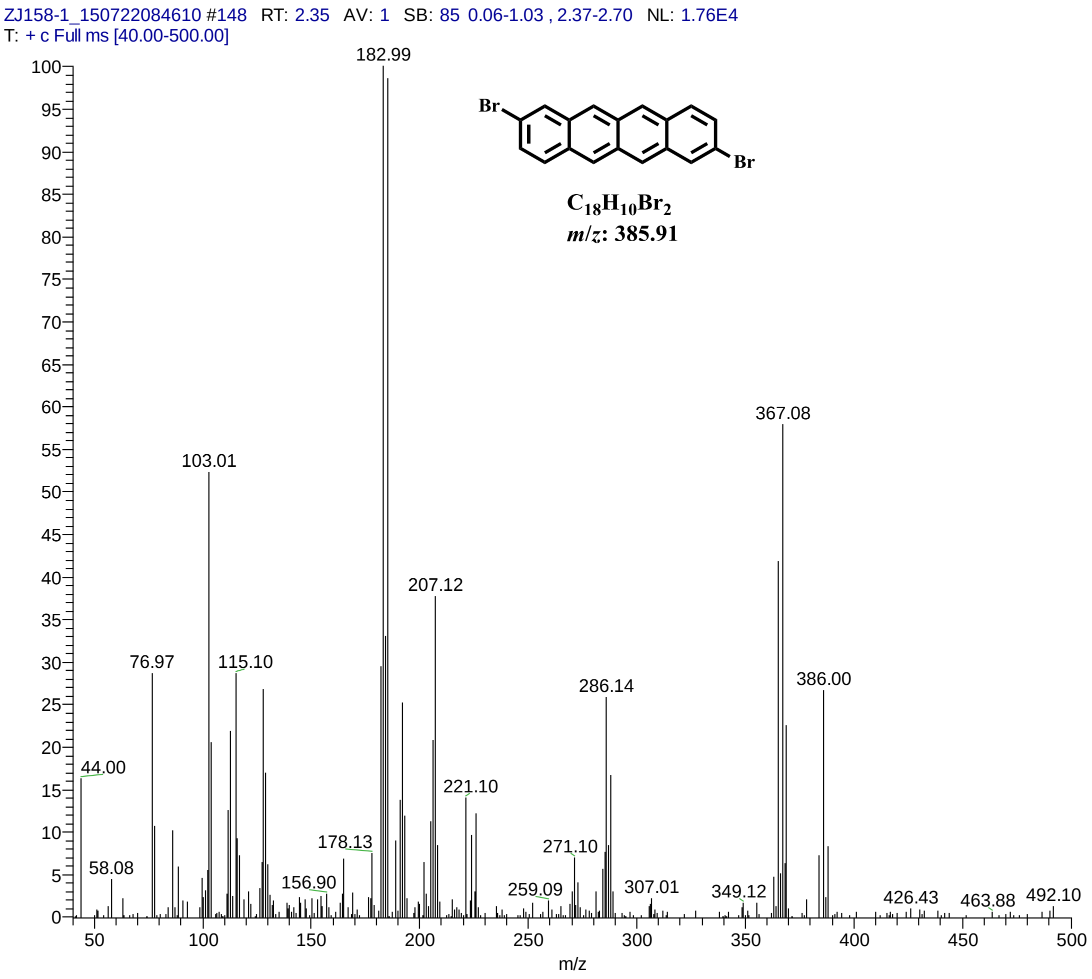

Fig. S12 MS (EI) of compound **2d**

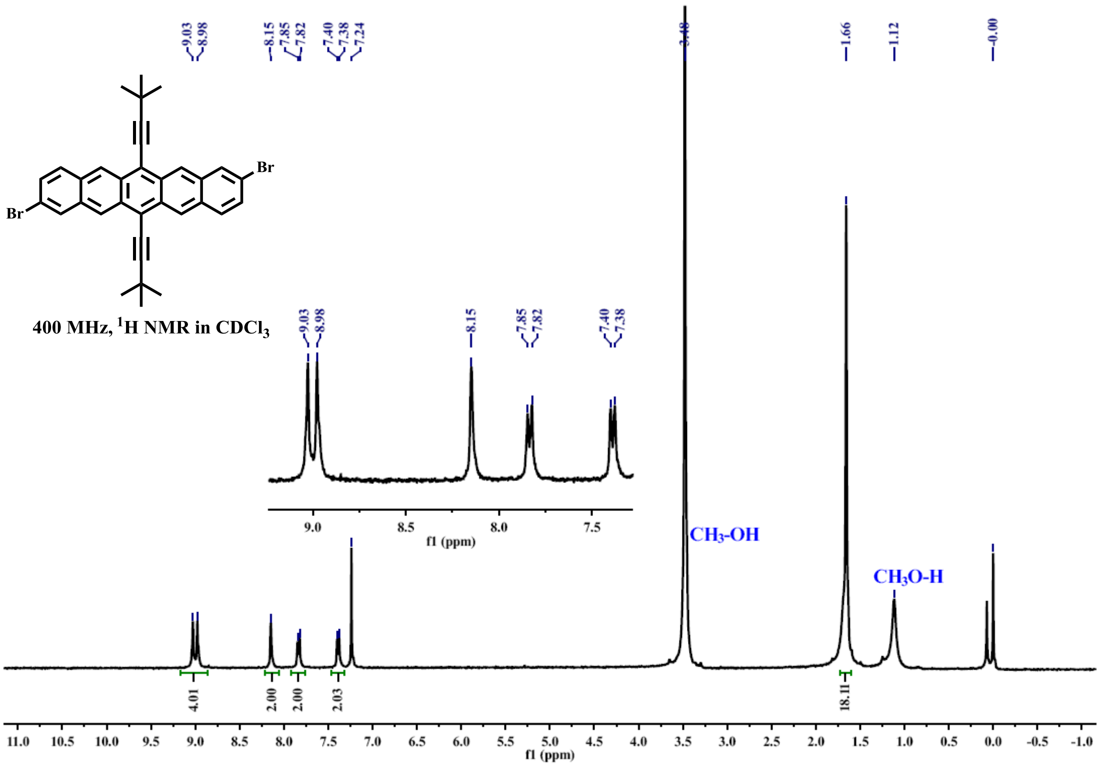

Fig. S13 ^{1}H NMR of compound **2e** (400 MHz, $CDCl_3$)

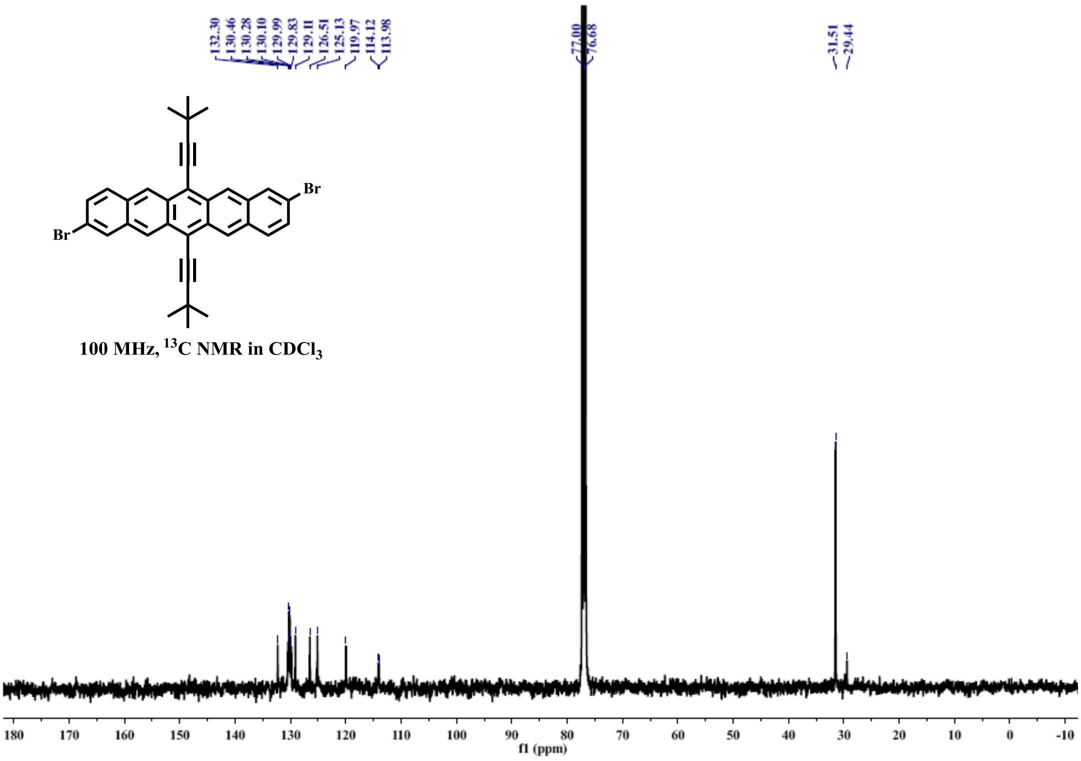

Fig. S14 ^{13}C NMR of compound **2e** (100 MHz, $CDCl_3$)

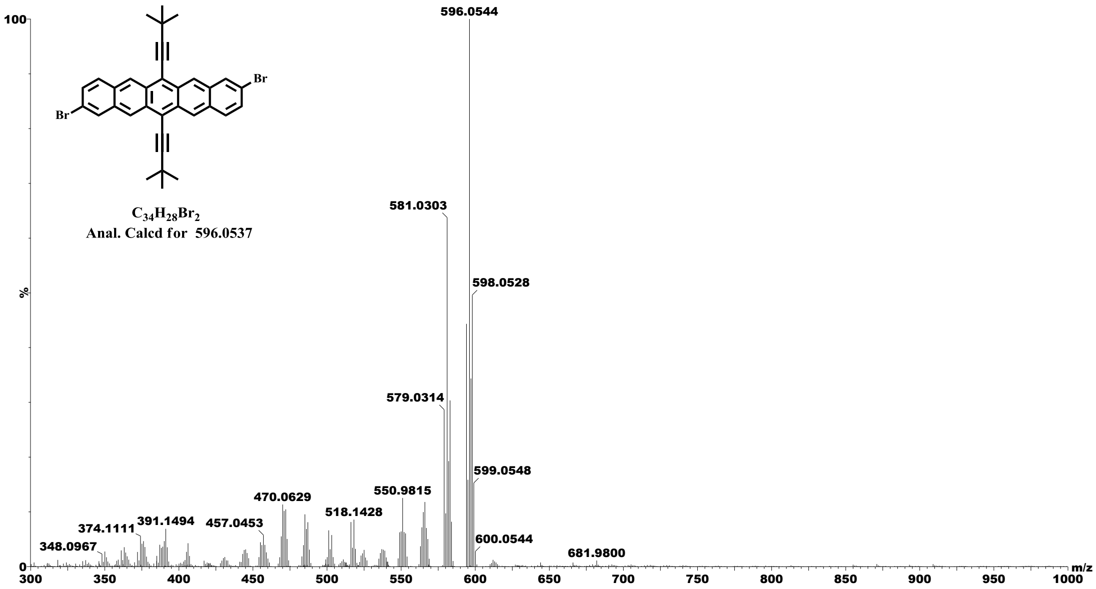

Fig. S15 HRMS (ESI) of compound **2e**

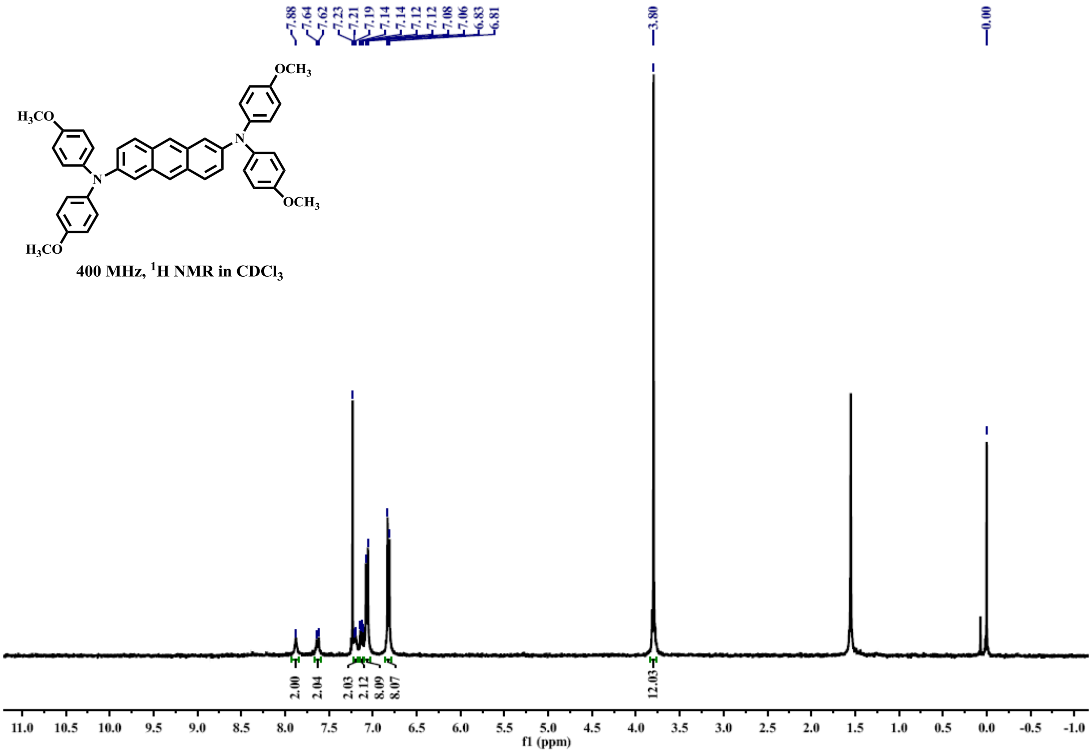

Fig. S16 ^{1}H NMR of compound **1c** (400 MHz, $CDCl_3$)

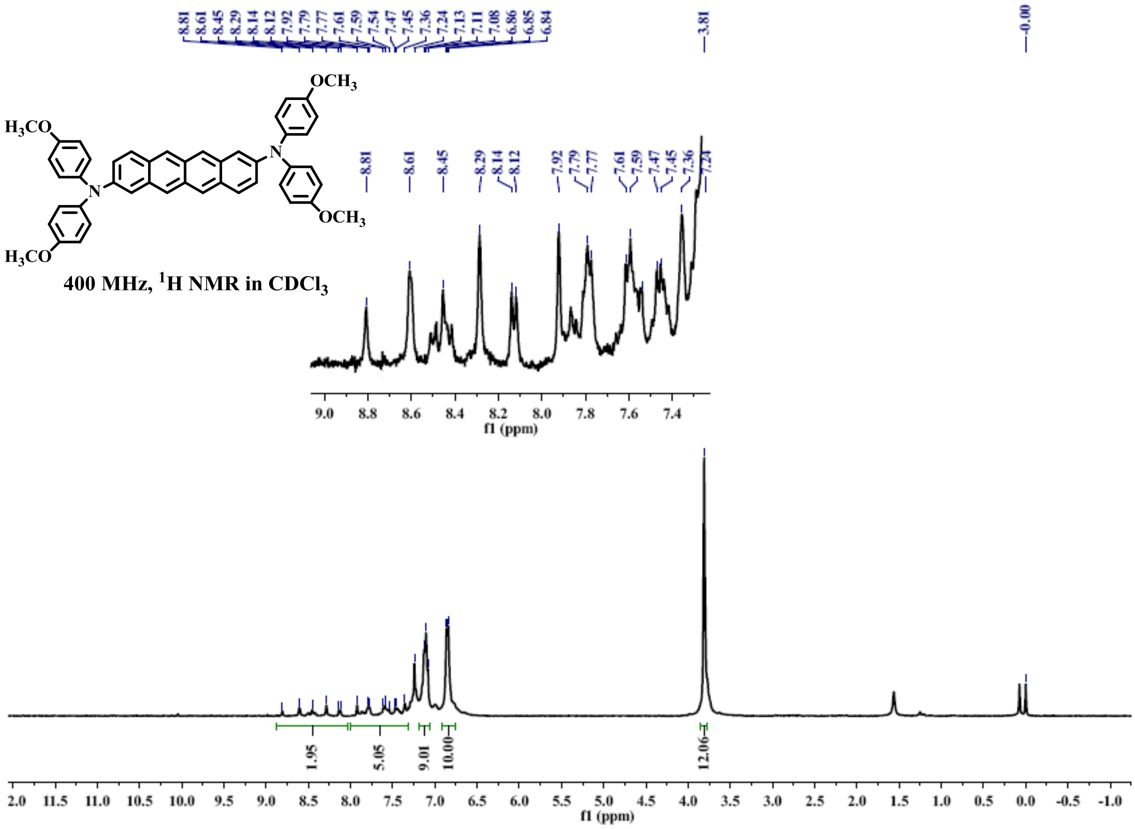

Fig. S17 ^{1}H NMR of compound **1d** (400 MHz, $CDCl_3$)

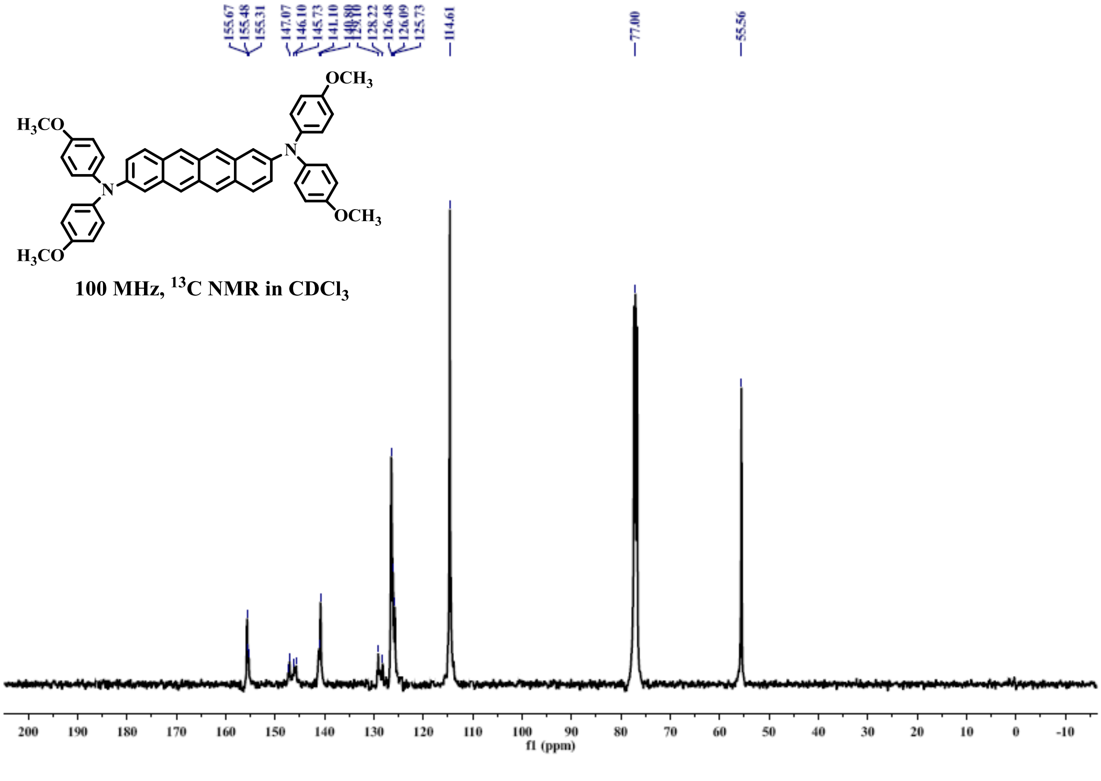

Fig. S18 ^{13}C NMR of compound **1d** (100 MHz, $CDCl_3$)

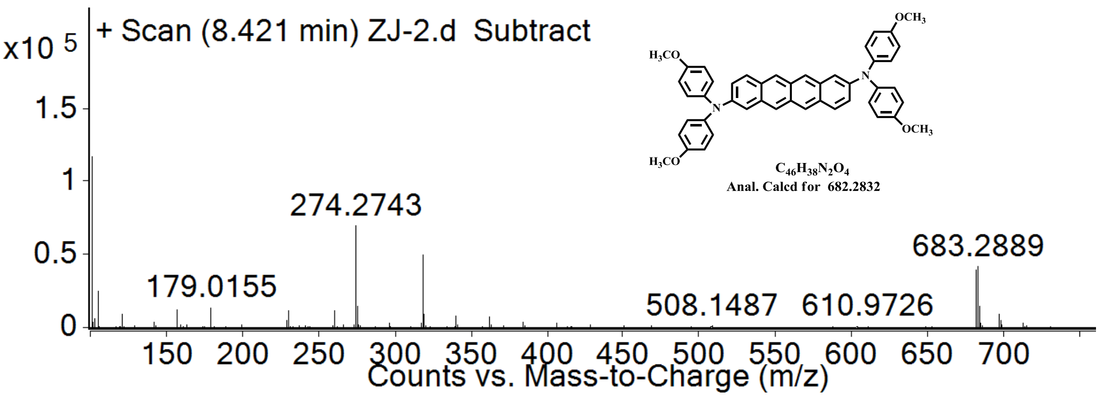

Fig. S19 HRMS (ESI) of compound **1d**

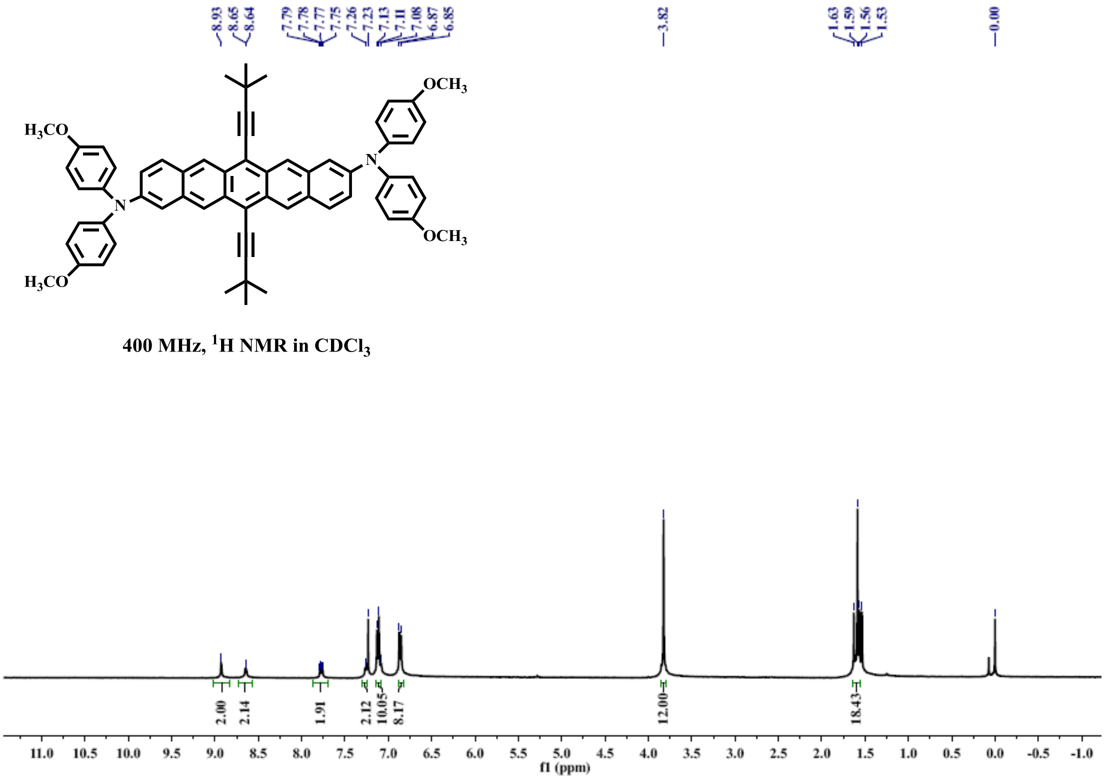

Fig. S20 ^{1}H NMR of compound **1e** (400 MHz, $CDCl_3$)

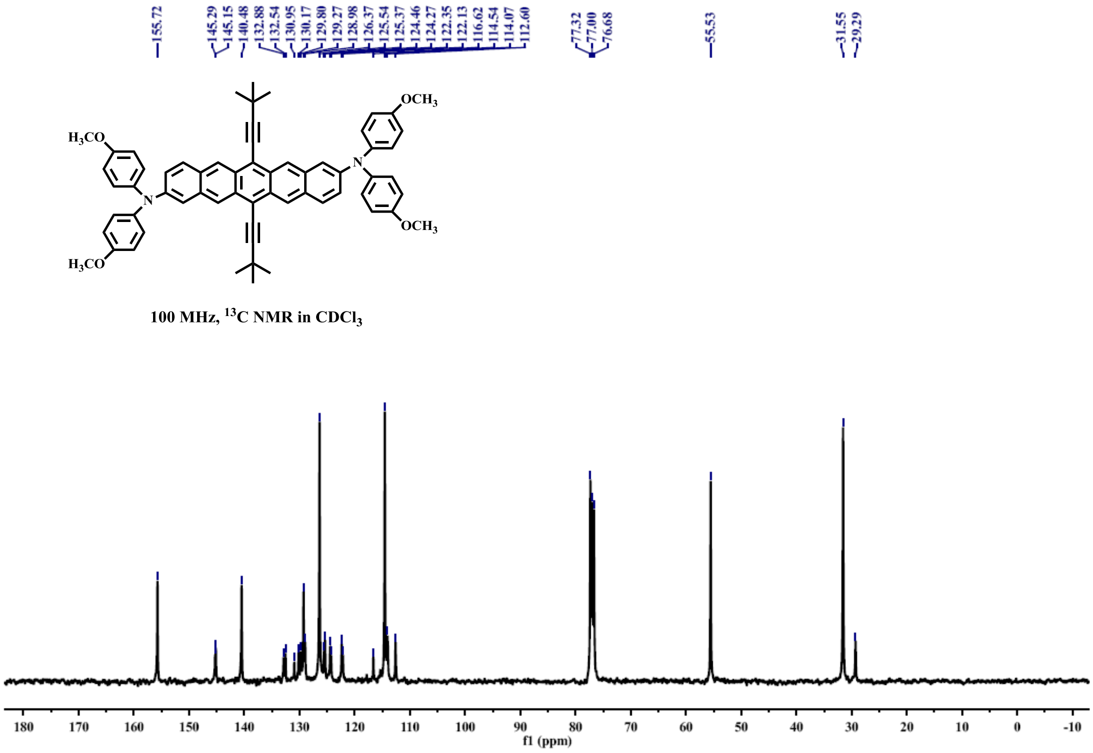

Fig. S21 ^{13}C NMR of compound **1e** (100 MHz, $CDCl_3$)

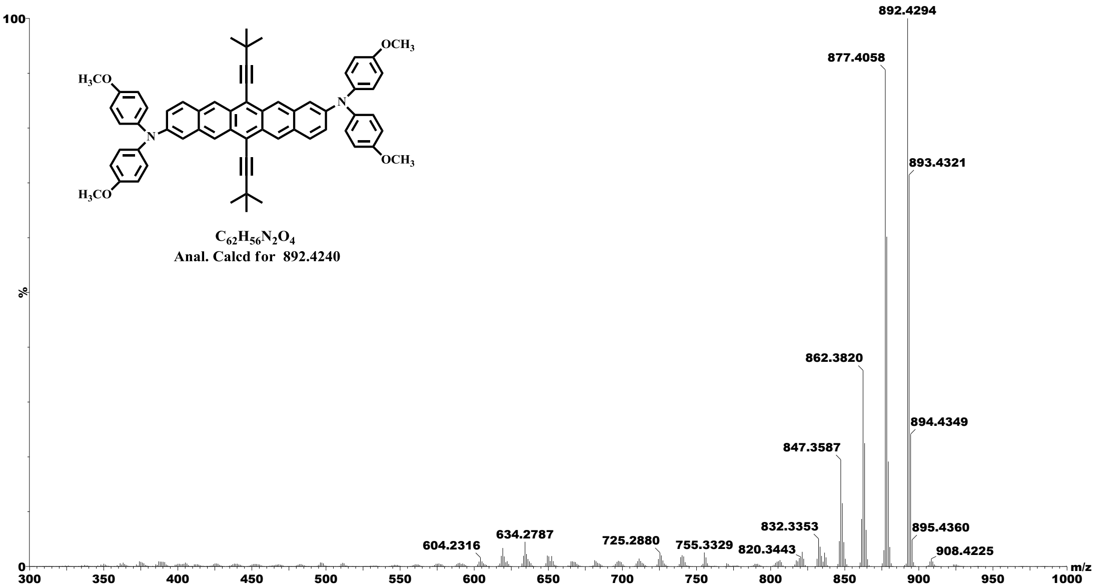

Fig. S22 HRMS (ESI) of compound **1e**
